# Organotypic brain slices as a model to study the neurotropism of the highly pathogenic Nipah and Ebola viruses

**DOI:** 10.1099/jgv.0.002038

**Published:** 2024-10-28

**Authors:** Michelle Gellhorn Serra, Lars Meier, Lucie Sauerhering, Jochen Wilhelm, Alexandra Kupke

**Affiliations:** 1 Philipps University Marburg, Institute of Virology, Marburg, Germany; 2 German Center for Infection Research (DZIF), TTU Emerging Infections, Giessen, Germany; 3 Department of Internal Medicine, Universities of Giessen and Marburg Lung Center, Member of the German Center for Lung Research, Giessen, Germany; 4 Institute for Lung Health, Cardio-Pulmonary Institute (CPI), Giessen, Germany

**Keywords:** choroid plexus, chemokines, cytokines, EBOV, neurotropism, NiV, organotypic brain slices

## Abstract

Nipah virus (NiV) and Ebola virus (EBOV) are highly pathogenic zoonotic viruses with case fatality rates of up to 90%. While the brain is a known target organ following NiV infection, involvement of the central nervous system in EBOV-infected patients only became more evident after the West African epidemic in 2013–2016. To gain a deeper comprehension of the neurotropism of NiV and EBOV with respect to target cells, affected brain regions and local inflammatory responses, murine organotypic brain slices (BS) were established and infected. Both NiV and EBOV demonstrated the capacity to infect BS from adult wt mice and mice lacking the receptor for type I IFNs (IFNAR^−/−^) and targeted various cell types. NiV was observed to replicate in BS derived from both mouse strains, yet no release of infectious particles was detected. In contrast, EBOV replication was limited in both BS models. The release of several pro-inflammatory cytokines and chemokines, including eotaxin, IFN-γ, IL-1α, IL-9, IL-17a and keratinocyte-derived chemokine (KC), was observed in both virus-infected models, suggesting a potential role of the inflammatory response in NiV- or EBOV-induced neuropathology. It is noteworthy that the choroid plexus was identified as a highly susceptible target for EBOV and NiV infection, suggesting that the blood–cerebrospinal fluid barrier may serve as a potential entry point for these viruses.

**Impact Statement**

Nipah virus (NiV) and Ebola virus (EBOV) are classified as highly pathogenic viruses, with case-fatality rates that are notably high. Individuals who have survived either NiV infection or Ebola virus disease frequently experience long-term neurological sequelae, which represent a significant burden not only for the survivors themselves but also for the local healthcare systems. The present manuscript describes our established adult mouse *ex vivo* organotypic brain slice culture model, which is designed to facilitate a deeper understanding of the neuropathology associated with NiV and EBOV. This model can serve as a valuable complement to the findings of animal studies and is aligned with the 3R principle, which aims to reduce, refine and replace animal experiments. Additionally, it offers insights into brain-specific inflammatory responses to EBOV and NiV infection and can be utilized to assess the efficacy of potential therapies aimed at reducing neuropathology.

## Introduction

Viral encephalitis represents a significant public health concern, with a considerable burden on our society, including hospitalization, disability and mortality. The high case-fatality rate is, in part, due to the lack of effective therapies, such as specific antivirals. Among the most common pathogens causing viral encephalitis are herpes simplex virus (HSV), Japanese encephalitis virus, tick-borne encephalitis virus and West Nile virus (WNV) [1,3]. Recently, there has been growing recognition of the neuropathogenic potential of several emerging viruses [4,5]. A considerable number of viruses within the *Mononegavirales* order have been identified as having the potential to cause neuropathological issues. Examples of these include the zoonotic and highly pathogenic Nipah virus (NiV) and Ebola virus (EBOV), which belong to the *Paramyxoviridae* and *Filoviridae* families, respectively. One of the primary target organs affected by NiV infection is the brain, often resulting in encephalitis [6,7]. However, for EBOV, neurological manifestations were not frequently observed prior to the West African epidemic in 2013–2016. During the epidemic, signs of acute or late-onset neurological dysfunction became more evident, even months after recovery [8,9].

The emergence of NiV in 1998/1999 resulted in an outbreak of severe viral encephalitis among pig farmers in Malaysia and abattoir workers in Singapore. As a result, nearly a million pigs were culled and there were 276 human cases, with a case-fatality rate of ~38% [10,11]. The majority of patients (~70%) exhibited the typical neurological symptoms, including drowsiness, headache, disorientation and confusion. Additionally, segmental myoclonus, hypoflexia and seizures were observed, followed by rapid deterioration of consciousness that progressed to coma and ultimately resulted in death [12]. Moreover, pulmonary involvement is commonly observed, particularly in outbreaks caused by the NiV-Bangladesh strain, which causes almost yearly outbreaks in Bangladesh and India with case-fatality rates ranging from 40 to 91% [13,14]. Autopsies of patients from the Malaysian outbreak revealed the presence of viral inclusions in neurons, as well as extensive microinfarction in the brain resulting from vasculitis-induced thrombosis and syncytial cell formation [15].

EBOV was first identified in 1976, following two almost simultaneous outbreaks of haemorrhagic fever in South Sudan and in Zaire (presently the Democratic Republic of Congo). The Sudan outbreak, caused by the Sudan virus (formerly Sudan ebolavirus), resulted in 284 cases with a case-fatality rate of 53%. In Zaire, the outbreak was caused by the Mayinga strain of EBOV (formerly Zaire ebolavirus), with 318 cases reported and a case-fatality rate of 88% [16,17]. Since the discovery of EBOV, isolated and sporadic outbreaks have been documented in Central Africa [18]. The largest outbreak of Ebola virus disease (EVD) occurred between 2013 and 2016 in West Africa, specifically in Guinea, Liberia and Sierra Leone. It resulted in over 28 000 cases and 11 000 fatalities. The clinical presentation of EVD typically manifests initially as flu-like symptoms, including fever, headache and muscular and joint pain. Furthermore, symptoms indicative of gastrointestinal involvement, such as nausea, vomiting and diarrhoea, may also be present. Respiratory symptoms, such as a sore throat, cough and shortness of breath, as well as cardiovascular involvement, resulting in oedema, rash and bleeding, are commonly observed. Neurological symptoms, including headache, confusion, delirium and coma, have also been documented [16,17]. EVD is typically an acute disease, but cases of persistence or relapse have been observed [19,21]. A well-documented example of EVD relapse is the case of a Scottish nurse who developed meningoencephalitis 9 months after recovery and was found to have infectious viral particles in her cerebrospinal fluid (CSF) [19].

The high pathogenicity of EBOV and NiV has resulted in a paucity of information regarding the route of infection, target cells and cell type-specific inflammatory responses within the central nervous system (CNS). Both NiV and EBOV are classified as biosafety level (BSL)-4 pathogens and are included in the World Health Organization’s R&D Blueprint initiative due to their potential to cause significant harm to public health care systems, given their epidemic potential and lack of effective countermeasures. In humans, brain endothelial cells appear among the initial target cell populations for NiV. Necrotic foci containing viral antigens have been observed in neurons and glial cells, frequently situated in close proximity to small blood vessels [12]. Furthermore, the paucity of autopsies conducted on EVD patients has resulted in a dearth of knowledge regarding EBOV brain pathology in humans. In a non-human primate (NHP) model of EBOV infection, the EBOV-VP40 antigen was observed in neurons, as well as in endothelial cells, monocytes and microglia [22]. This underscores the extensive range of possible target cells for EBOV, although it should be noted that these observations pertain to the terminal stages of infection. It is noteworthy that wt C57BL/6 J mice are not susceptible to either NiV or EBOV infection. However, mice lacking the receptor for type I IFNs (IFNAR^−/−^) rapidly succumb to either virus upon infection, exhibiting disease progression and clinical features that are similar to those observed in humans and NHPs [23,25].

Culturing of brain tissue represents an efficacious methodology for investigating neurological pathologies such as viral encephalitis. The cellular composition, 3D architecture, interactions between different cell types and functions of brain tissue can be maintained in explanted brain slices (BS), which retain many relevant *in vivo* conditions [1]. The most prevalent method for culturing *ex vivo* organotypic BS, typically isolated from embryonic or neonatal animals, is the cultivation of explanted brains on a porous membrane in an air–liquid interface [26]. Organotypic BS have been successfully employed by numerous researchers to investigate viral infections *ex vivo*. In many cases, these models have been observed to reproduce the *in vivo* pathogenesis that is known to occur during viral encephalitis [1]. In the case of measles virus (MV) and the canine distemper virus (Paramyxoviruses), BS have been employed to assess neurovirulence and neurotropism [27]. Utilizing BS, the role of neuroinflammatory responses during WNV infection, particularly of microglia, has been analysed [28]. Furthermore, the susceptibility and the inflammatory environment induced by Rift Valley fever virus have been recently investigated using BS [29]. Additionally, the role of apoptosis of brain immune cells in limiting type I IFN responses during HSV type I infection has also been analysed using BS [30].

In this study, we sought to ascertain the susceptibility of explanted BS to either NiV or EBOV infection. To this end, BS from adult IFNAR^−/−^ or wt C57BL/6 J mice (aged between 3 months and 1.5 years) were used to investigate target cells, virus spread and brain-specific inflammatory responses. The objective was to gain a deeper understanding of the observed differences in their susceptibility to NiV and EBOV *in vivo* between the two mouse strains. Consequently, the role of type I IFNs in virus susceptibility, cytokine and chemokine release and tissue damage was investigated. The suitability of the model for infection with neurotropic viruses was confirmed under BSL-2 conditions, using the strong neurotropic vesicular stomatitis virus (VSV), a Rhabdovirus within the *Mononegavirales* order that can infect numerous cells of the brain, including endothelial cells and neurons [31,32]. NiV and EBOV were capable of infecting BS from wt and IFNAR^−/−^ mice, although differences were observed in virus replication. Notably, the choroid plexus displayed a high susceptibility to both EBOV and NiV infections, suggesting that the blood–CSF barrier could serve as a potential entry point. Furthermore, both viruses triggered the release of numerous pro-inflammatory cytokines and chemokines, indicating a potential involvement of the inflammatory response in NiV- or EBOV-induced neuropathology.

## Methods

### Animals

Type I IFN receptor knock-out (IFNAR^−/−^) mice [33] on a C57BL/6J background were generously provided by U. Kalinke (Twincore, Hannover, Germany) and bred at the Philipps University of Marburg. Wt mice of the same background were purchased from Charles River (Sulzfeld, Germany). Male and female adult mice aged between 3 months and 1.5 years were used in this study.

### Isolation and cultivation of adult mouse organotypic BS

The organotypic BS culture was established based on different published protocols [26,34] and modified for our own purposes. Briefly, adult mice were anaesthetized with isoflurane (AbbVie) and subsequently euthanized by cervical dislocation. The brains were isolated and placed in ice-cold dissection buffer [100 ml Hanks' Balanced Salt Solution (HBSS; Gibco), 2.24 ml d(+)-glucose (Sigma-Aldrich), 500 µl penicillin/streptomycin (10 000 U ml^−1^; Gibco) and 1 ml 100 mM kynurenic acid (Tocris)] where the cerebellum was removed and the remaining tissue bisected along the midline. The hemibrains were cut coronally with a McIlwain Tissue Chopper (Stoelting Co.) into 350 µm-thick slices. The slices were carefully separated and transferred with a cut plastic Pasteur pipette onto Millicell (MilliporeSigma) culture inserts in six-well culture plates containing 1 ml neuroglial (NG)-Medium [50 ml Neurobasal medium (ThermoFisher), 4 ml N-2-hydroxyethylpiperazine-N-2-ethane sulfonic acid (HEPES; Gibco), 500 µl GlutaMAX (Gibco), 100 µl gentamicin (ThermoFisher), 1 ml B27-Supplement (Gibco)] with four slices per culture insert (olfactory bulb, frontal, middle and caudal sections) (Fig. S1, available in the online version of this article). The transferred dissection buffer covering the slices was removed. Two hours after plating, 1 day after isolation and subsequently every third day, the cell culture medium was exchanged. BS were incubated at 37 °C with 5% CO_2_ in a humidified incubator. Infection studies were performed 4 days after isolation.

### MTT viability assay

The viability of the cultured BS was assessed using the MTT assay. To perform this assay, the yellow tetrazolium salt MTT (thiazolyl blue tetrazolium bromide; Sigma Aldrich) was added to the culture media at a concentration of 0.5 mg ml^−1^ and incubated at 37 °C. The slices were examined 3 h after this procedure and on the following day. The viability of the slices was documented by photographing the slices. The living tissue metabolizes MTT into a purple formazan and displays a blue-purple colouration, while the non-living tissue shows no colouration.

### Lactate dehydrogenase viability assay

Lactate dehydrogenase (LDH) viability assays were used to evaluate tissue damage resulting from infection, as well as to analyse the recovery of the cultured slices after the slicing process. LDH is a ubiquitous enzyme present in the cytoplasm of cells. Upon damage to the plasma membrane, it is readily released into the culture media. The release of LDH is frequently employed as an indicator of cellular and tissue damage [35]. To achieve this, the LDH-Cytotoxicity Assay Kit II (Merck) was used following the manufacturer’s instructions. Briefly, an LDH Reaction Mix was prepared by combining LDH Assay Buffer with a water-soluble tetrazolium salt to obtain a 2% solution. Afterwards, 10 µl of cell-free supernatant samples were added in triplicates into clear 96-well plates. A background control and a positive control were prepared by giving the 10 µl fresh culture medium in triplicate or 5 µl LDH onto the corresponding wells, respectively. Next, the 100 µl LDH Reaction Mix was administered rapidly with a multichannel pipette to each sample and control and mixed. The assay was incubated for 30 min at room temperature (RT) protected from light. Lastly, the assay was analysed by measuring the absorbance at 450 nm using a PHOmo Microplate Reader (Autobio Diagnostics Co.).

### Viruses

Stocks for EBOV-Mayinga (NC_002549) and NiV-Malaysia (isolate described previously [36]) used for all experiments were produced on VeroE6 and Vero76 cells (CVCL_0574, CVCL_0603), respectively. Supernatants were purified via ultracentrifugation layered with 20% sucrose and 50% tissue culture infectious dose (TCID_50_)-titered after one cycle of freezing and thawing. Stocks for VSV-Indiana (kindly provided by Prof. Dr. Friedemann Weber) were produced in VeroE6 and were TCID_50_-titered accordingly.

### Viral infection

The viral inoculum, comprising 1×10^6^ TCID_50_ (NiV and VSV) and 1×10^5^ TCID_50_ (EBOV) of virus mixed with PBS to a total volume of 20 µl, was added dropwise on top of each slice. Mock inoculations were performed in the same manner, utilizing PBS alone. Infection was analysed at 1, 24, 48, 72 and 96 hpi.

### Immunofluorescence analysis

Organotypic BS were fixed in 4% paraformaldehyde (PFA) for a minimum of 3 h for characterization experiments and VSV infections or twice for 24 h for infections performed in the BSL-4 laboratory (NiV and EBOV) and stained based on a published protocol [37]. In brief, following fixation, slices were removed from the membrane by carefully pipetting PBS near the slices and then transferred with a cut plastic pipette into a 24-well plate. PBS was then removed and replaced by 0.5% Triton-X (Sigma-Aldrich) in PBS and incubated overnight at 4 °C to permeabilize the tissue. To prevent non-specific antibody binding, a solution of 20% BSA (Serva) in PBS was prepared and used for blocking for at least 4 h at RT or overnight at 4 °C. The slices were then incubated with primary antibodies diluted in 5% BSA in PBS at least 4 h at RT or overnight at 4 °C in a humid chamber. After washing, slices were incubated with fluorophore-coupled secondary antibodies and with DAPI in a humid chamber for at least 4 h and washed sequentially with PBS and H_2_O. Finally, the slices were mounted on slides with Fluoroprep (bioMérieux). Images and image stacks were captured on a confocal laser scanning microscope (Leica TCS SP5 II) and converted into 2D images (maximum intensity Z-projection) as well as 3D images with Fiji software (open source [38]). Mouse anti-βIII-tubulin (MA1-118; Thermo Fisher Scientific), rat anti-βIII-tubulin (T2200; Sigma-Aldrich) or chicken anti-microtubule associated protein 2 (MAP2; PA1-16751; Thermo Fisher Scientific) were used as markers for neurons. Rat anti-glial fibrillary acidic protein (GFAP; 13-0300; Thermo Fisher Scientific) or rabbit anti-GFAP (PA5-16291; Thermo Fisher Scientific) was used to identify astrocytes. Rat anti-myelin basic protein (MBP; ab7349; Abcam) or chicken anti-MBP (PA1-10008; Thermo Fisher Scientific) was used as a marker for oligodendrocytes. Rabbit anti-ionized calcium-binding adapter molecule 1 (Iba1; 019-19741; Wako Chemicals) and rat anti-CD31 (557 355; BD Biosciences) were used as markers for microglia and endothelial cells, respectively. The presence of infection with VSV, NiV and EBOV was detected with mouse anti-VSV-N (10G4; Kerafast Inc.), rabbit anti-NiV-M (IG1321; ImmunoGlobe GmbH) and chicken anti-EBOV-NP (kindly provided by R. Schade, Charité Medical School of Berlin, Berlin, Germany [39]). For double staining, parallel utilization of primary antibodies from different species was employed. Secondary antibodies targeting the species of the primary antibody coupled with either Alexa Fluor 488 or 594 and DAPI to visualize the nuclei were used.

### Real-time quantitative PCR

For real-time quantitative PCR (RT-qPCR) analysis, infected slices as well as culture supernatants were collected at various time points. Supernatants were collected and centrifuged to remove cell debris. Slices were transferred into lysing matrix tubes (MP Biomedicals) containing 1 ml Dulbecco's Modified Eagle Medium (DMEM; Gibco), homogenized with a Mixer Mill MM 400 (Retsch) and centrifuged at 2400 r.p.m. for 5 min to remove debris. For viral RNA isolation, the QIAamp Viral RNA Mini Kit (QIAGEN) or the RNeasy RNA mini kit (QIAGEN) was used according to the manufacturer’s protocol. The isolated viral RNA was reverse transcribed and multiplied with the One-Step RT-qPCR Kit (QIAGEN) with specific primers and probes for VSV-N [40], NiV-NP [41] and EBOV-GP [42]. Specific standards (10^10^–10° copies µl^−1^) were prepared as previously described [41] and used for virus quantification. The lower limit of detection (LOD) was defined as the last standard detected below cycle threshold (*C*_t_) 40. For the supernatant RT-qPCR, the LOD is equivalent to 10°copies µl^−1^ for NiV and 10^1^ copies µl^−1^ for EBOV and VSV. The LOD for the homogenate RT-qPCRs varies depending on the RNA concentration of each sample.

### TCID_50_ assay

To determine infectious titres in infected BS and virus stocks, TCID_50_ assays were performed. For this, Vero76 (CVCL_0603) cells for NiV infections or VeroE6 (CVCL_0574) cells for EBOV or VSV infections were seeded on 96-well plates at a density of 50–60%. Virus stocks, cell-free supernatants or tissue homogenates were serially diluted and incubated for 3–7 days at 37 °C. For BS supernatants and homogenates, a minimum of four replicates at fivefold or tenfold dilutions, and for virus stocks, a minimum of eight replicates at tenfold dilutions were used. The cytopathic effect was microscopically analysed and titres were calculated using the Spearman–Kärber method [43].

### *In-situ* hybridization

To identify the infected areas of the brain, *in-situ* hybridization (ISH) was performed using RNAscope reagents (2.5 HD Detection Reagents – RED, 322360; Target Retrieval Reagents, 322000; Hydrogen Peroxide+Protease Plus, 322330; Wash Buffer Reagents, 310031) and probes against EBOV (V-EBOV-VP35-GP, 493261) and NiV (V-Nipah-StrM.B.-N, 439251) detecting viral mRNA and antigenome were used as described previously [44,45]. Briefly, infected BS were fixed in formalin, embedded in paraffin and resliced for histological analyses. The slides were baked at 60 °C, deparaffinized with xylene and 100% ethanol and pretreated with RNAscope Pretreatment Reagents. Antisense probes were then hybridized to viral sense RNA in a humidity tray for 2 h at 40 °C. To enhance the signal, a multi-step process was performed using a preamplifier and several amplifiers with binding sites for alkaline-phosphatase-labelled probes. The signal was detected by the addition of Fast Red substrate. Subsequently, the slides were counterstained with Gill’s Hematoxylin I and 0.02% ammonia water. In parallel, an RNAScope Negative Control Probe (310043) was used to control background staining.

### Luminex assay

BS supernatants collected at different time points after infection were screened for cytokine and chemokine release with the Bio-Plex Pro Mouse Cytokine 23-Plex Assay (BioRad Laboratories) according to the manufacturer’s instructions. Briefly, beads were transferred to the assay plate and washed two times. Standards, samples and controls were added and incubated for 30 min, followed by 25 µl detection antibody solution incubated for 30 min and 50 µl streptavidin-phycoerythrin (PE) for 10 min. Finally, 125 µl assay buffer was used to resuspend the samples for 30 s, and data were acquired on a BioPlex 200 system (BioRad Laboratories). Incubation steps were performed on a shaker at 850 r.p.m. and RT. Washing steps were carried out after each incubation step on a Bio-Plex Pro Wash Station (BioRad Laboratories). BSA was added to serum-free samples to 0.5% final w/v. The analysed cytokines included IL-1α, IL-1β, IL-2, IL-3 IL-4, IL-5, IL-6, IL-9, IL-10, IL-12 (p40), IL-12 (p70), IL-13, IL-17A, eotaxin, IFN-γ, TNF-α, keratinocyte-derived chemokine (KC), regulated upon activation normal T cell expressed and secreted (RANTES), macrophage inflammatory protein (MIP)-1α, MIP-1β, monocyte chemotactic protein-1 (MCP-1), granulocyte colony-stimulating factor (G-CSF) and granulocyte-macrophage colony-stimulating factor (GM-CSF). To calculate concentrations, a five-parameter logistic regression was used. The lower limit of quantification and upper limit of quantification were defined as the lowest standard and highest standard for each analyte, respectively.

### Statistical analysis

Data were analysed by general linear models using log-transformed values. The reasonability of model assumptions was checked by residual diagnostics. Repeated-measures data were analysed using general linear mixed models including a random intercept term for the animal ID. Statistical significance of effects of time (hpi), differences between treatments (mock or virus infection) and treatment differential time effects (time–treatment interaction) were assessed by Wald tests of the respective coefficients in the linear model. Data from the Luminex assays with a significant proportion of values below the lower limit of quantification were analysed using tobit models with values being left censored at the lower limit of quantification.

All analyses were performed in R 4.4.1 R [46]. Mixed models were fit using the package lmerTest 3.1–3 [47] and tobit models were fit using the survreg function of the package survival 3.5-8 [48].

## Results

### Adult mice organotypic BS remain viable for a long period and contain main cell types of the brain

**Fig. 1.**
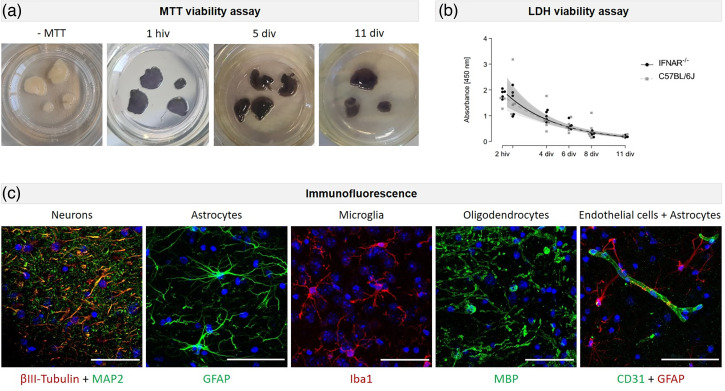
Characterization of murine adult organotypic BS. (a) Viability was analysed by the MTT assay at 1 h and 5 and 11 days after isolation. Purple colouration represents viable tissue. As a control, BS were incubated without MTT (-MTT), *n*=3. (**b**) The LDH viability assay showing LDH release over time. Slice media from IFNAR^−/−^-BS or C57BL/6J-BS were collected and analysed at different time points to assess tissue damage. Individual values are shown. A regression model was fitted to describe an exponential relationship between absorbance and time in culture, with log-normal errors. The curves depict the model predictions, with the shaded areas representing the approximate 95% confidence bands. Note that the confidence bands of the two models largely overlap. (**c**) Analysis of BS cellular composition via IF. The main cell types of the brain were identified: neurons (βIII-tubulin, MAP2), astrocytes (GFAP), microglia (Iba1), oligodendrocytes (MBP) and endothelial cells (CD31). Cellular markers are shown in red and green. Nuclear staining: DAPI (blue). Scale bars: 50 µm. div, days *in vitro*; hiv, hours *in vitro;* MTT, 3-(4,5-dimethylthiazol-2-yl)-2,5-diphenyltetrazolium bromide.

In the present study, we established BS from adult mice to analyse the susceptibility of fully developed brain tissue to NiV and EBOV infection. This approach was selected due to the potential for variability in the susceptibility of brain tissue across different developmental stages, including embryonic, neonatal and adult. For example, the expression of the cellular receptors for NiV ephrin B2 and B3 is known to change during brain development [49], which may result in differences in susceptibility. First, the viability of the cultured slices was assessed by means of MTT assays. BS exhibiting a purple colouration following incubation with MTT indicative of viable tissue was observed up to the latest analysed time point at 11 days post-isolation (Fig. 1a). As anticipated, the cutting process inevitably resulted in the severing of several axons and cell processes, which subsequently induced cell death. To ascertain whether the rate of cell death was reduced over time, a culture medium from IFNAR^−/−^-BS or C57BL/6J-BS was collected and analysed at different time points via a colourimetric LDH assay. An evident release of LDH was observed at 2 h and 1 day after isolation but exhibited a progressive decline over time in both IFNAR^−/−^ and wt tissue (*P*<0.001 each) (Fig. 1b). The rate of decrease was found to be comparable between BS derived from both mouse strains. Furthermore, the presence of the main cell types of the brain, including neurons, astrocytes, oligodendrocytes, microglia and endothelial cells (Fig. 1c), was analysed and confirmed via immunofluorescence (IF). These results demonstrate the viability of adult mouse BS, the maintenance of the cellular composition and 3D architecture (Fig. S2), despite the initial cell death and tissue injury caused by the slicing process. Accordingly, this model system was further employed to investigate viral cell tropism and spread, in addition to the induced brain-specific inflammatory responses to highly neurotropic viruses.

### Adult organotypic BS are susceptible to VSV

**Fig. 2.**
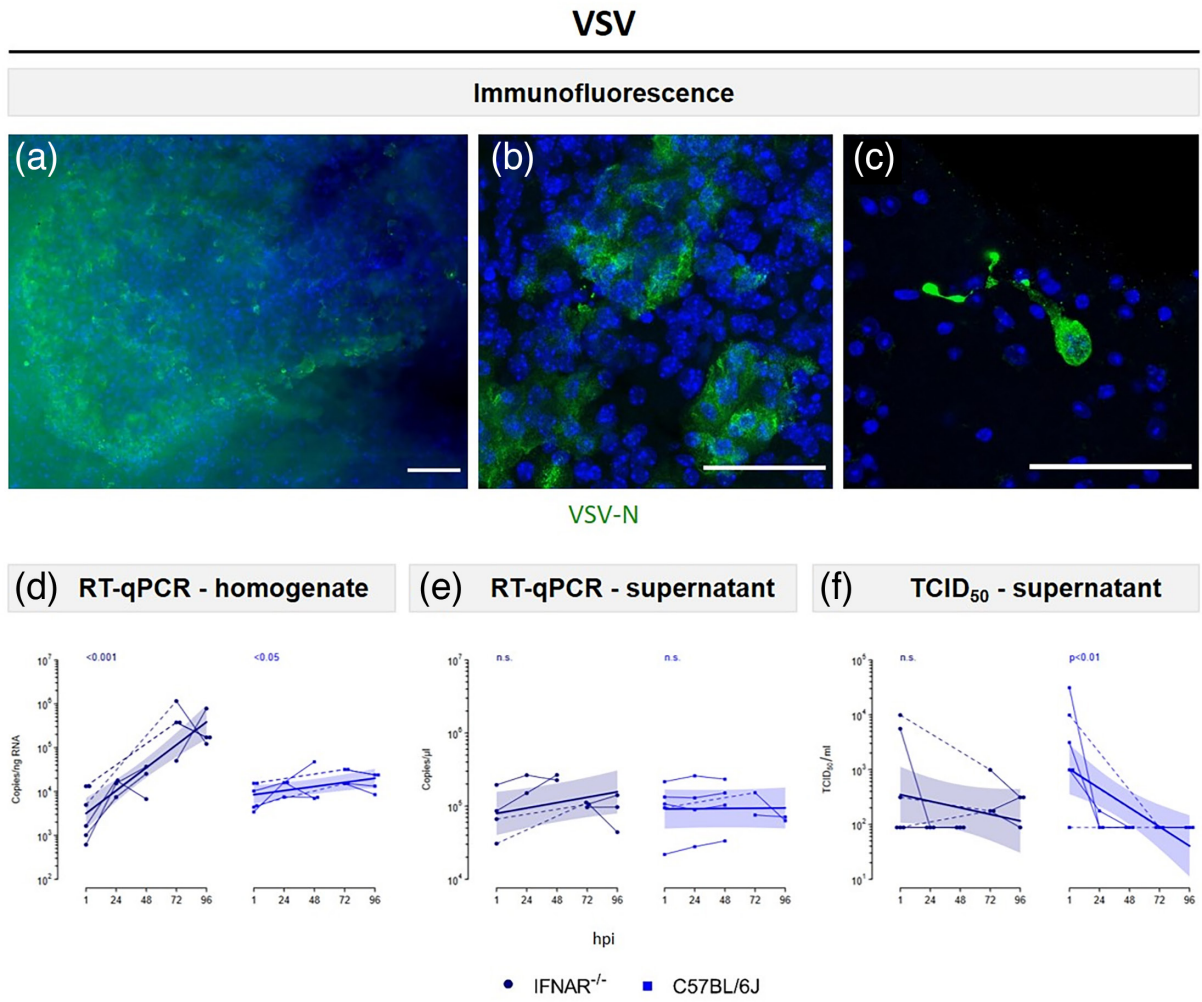
Susceptibility of murine adult organotypic BS to VSV. (a–c) IF of IFNAR^−/−^-BS infected with VSV at 24 hpi. Viral staining: VSV-N (green). Nuclear staining: DAPI (blue). Scale bars: 50 µm. (**d**) Real-time qPCR to quantify i.c. VSV RNA loads at different time points. (**e**) RT-qPCR to quantify e.c. VSV RNA loads at different time points. (**f**) TCID_50_ titration of supernatants at different time points to analyse the release of infectious particles. Data are presented as individual values with connecting lines between related experiments. Dotted lines indicate time intervals that are discontinuous. A regression model was fitted to describe an exponential relationship between concentration and hpi, with log-normal errors. The curves depict the model predictions, with the coloured areas representing the approximate 95% confidence bands. The *P*-values are shown above the curves and refer to Wald t-tests of the growth rate coefficient (see the Methods section for details). Values below the LOD were defined as 0.5×LOD. n.s., not significant.

In order to evaluate the susceptibility and the suitability of the model for the study of neurotropic virus infection, BS were infected with VSV, as a neurotropic model virus, under BSL-2 conditions. To this end, 1×10^6^ TCID_50_ ml^–1^ of virus was mixed with PBS to a total volume of 20 µl per slice and added dropwise on top of each slice. To ascertain whether VSV is capable of infecting the BS, an IF analysis was conducted. VSV-N (nucleoprotein) staining was observed in extensive areas of IFNAR^–/–^-BS (Figs 2a and S3), in scattered small foci (Fig. 2b) or in single infected cells of various morphologies. Additionally, cells with neuronal characteristics were observed, as determined by their round and prominent cell soma and long cell processes, presumably axons (Fig. 2c). Moreover, VSV-N staining was observed in C57BL/6J-BS, although to a lesser extent than in IFNAR^–/–^-BS (Fig. S4). Subsequently, the question was addressed as to whether BS could provide the necessary conditions for viral replication and progeny production. Viral replication was analysed via RT-qPCR, whereby the intracellular (i.c.) and extracellular (e.c.) viral RNA loads in tissue homogenates and supernatants, respectively, were quantified. An increase in i.c. viral RNA load over time was observed in both IFNAR^–/–^-BS (*P*<0.001) and C57BL/6J-BS (*P*<0.05) following VSV infection (Fig. 2d). It is noteworthy that clear differences in the growth rate were observed between the genotypes (*P*<0.001). These findings suggest that VSV was capable of replicating in both BS models, with a higher titre observed in BS-IFNAR^–/–^. No distinct trend in e.c. viral RNA load, representing viral release, over time could be detected in BS derived from either mouse strain (Fig. 2e). At 1 hpi, high titres of infectious particles representing the inoculum were detected in the supernatants via TCID_50_ assays. These levels subsequently declined over time, reaching levels below the LOD (Fig. 2f), indicating that no release of infectious particles was detected. These data prove the suitability of the model to study neurotropic viral infections and the susceptibility of the BS to VSV infection, especially in the context of impaired type I IFN signalling.

### NiV and EBOV are able to infect various cells of adult organotypic BS, especially cells of the choroid plexus

**Fig. 3.**
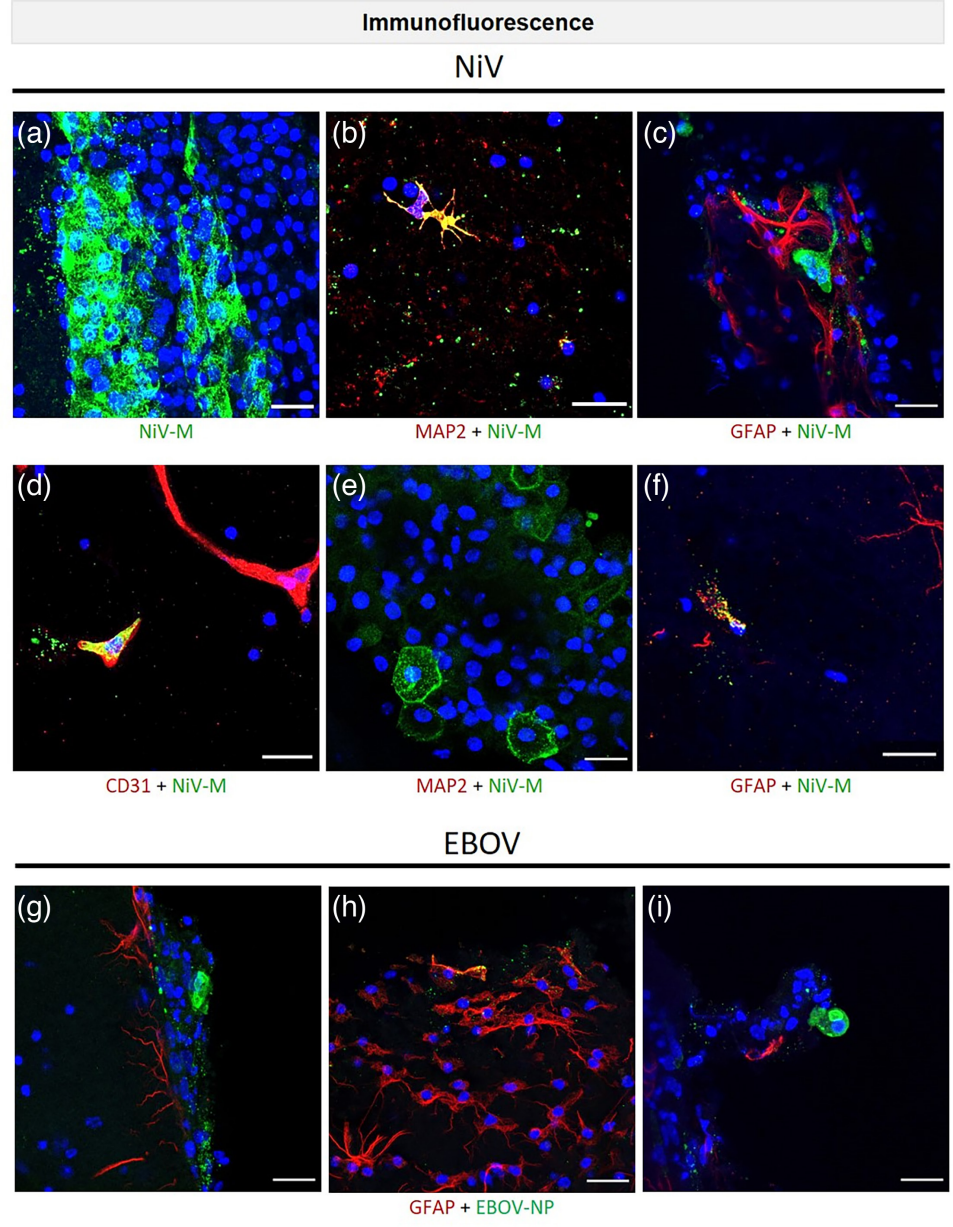
Susceptibility of adult organotypic BS to NiV and EBOV. (a–f) IF of NiV-infected IFNAR^–/–^-BS at different time points. (**g–i**) IF of EBOV-infected IFNAR^−/−^-BS at 48 hpi. Cell staining: endothelial cells (CD31, red), neurons (MAP2, red) and astrocytes (GFAP, red). Viral proteins: NiV-M (green) and EBOV-NP (green). Nuclear staining: DAPI (blue). Scale bar: 20 µm.

To analyse the susceptibility of BS to NiV or EBOV infection, IF and ISH were conducted to identify infected cell types and affected brain regions, respectively. Following infection with NiV at an inoculation dose of 1×10^6^ TCID_50_ ml^−1^, areas of extensive infection were observed (Fig. 3a). Among the infected cells were neurons (Fig. 3b), endothelial cells (Fig. 3d), cells of cuboidal morphology (Fig. 3e) and astrocytes (Fig. 3f). Moreover, the formation of small syncytia was observed (Fig. 3c). Furthermore, NiV-M (matrix protein) staining was observed to be distributed throughout infected cells, with a notable localization near the plasma membrane. Infection of BS with EBOV was conducted using an inoculation dose of 1×10^5^ TCID_50_ ml^−1^. IF analysis facilitated the identification of infected cells through double staining or based on cell morphology. Infected astrocytes as well as the presence of inclusion bodies in cells and the neuropil were observed (Fig. 3g–i). EBOV-NP (nucleoprotein) staining was typically observed in areas exhibiting astrogliosis (Fig. 3h). However, BS infected with both viruses often resulted in severe cell damage, which complicated the definitive identification of cell types.

**Fig. 4.**
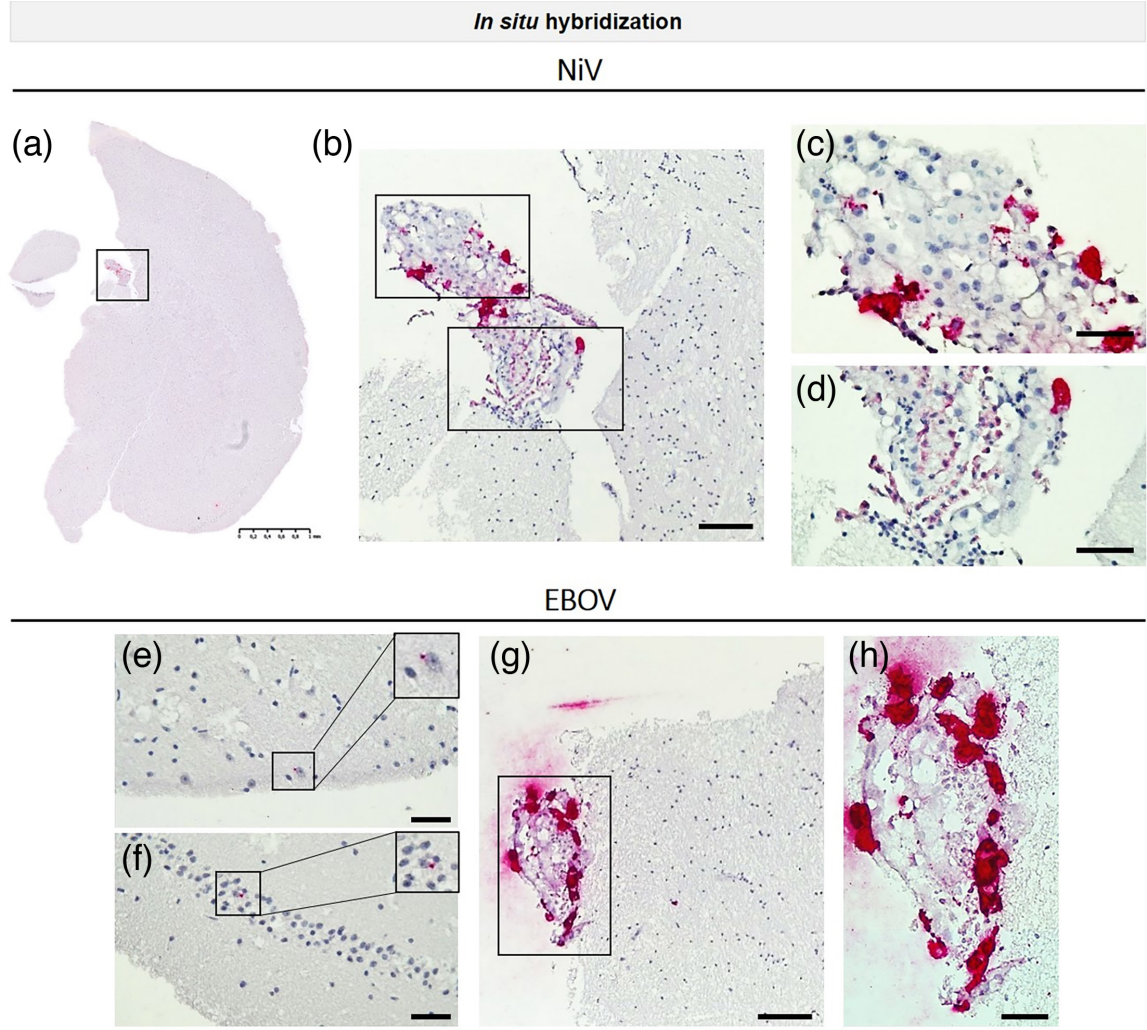
Susceptibility of different brain regions to NiV and EBOV. (**a**) ISH of a whole BS showing staining for positive stranded viral RNA (V-Nipah-Strain Malaysia/Bangladesh-N-Probe) within the choroid plexus of the lateral ventricle at 72 hpi in IFNAR^−/−^-BS. The entire slide was scanned using the NanoZoomer system from Hamamatsu. (**b–d**) Higher magnification pictures of (a). (**e–h**) ISH of EBOV-infected BS showing positive staining for viral RNA (V-EBOV-VP35-GP-probe) in neurons (e–f) and in the choroid plexus (g) at 96 hpi in IFNAR^–/–^-BS. (**h**) Higher magnification picture of (g). Scale bars: (a) 1 mm, (**b, g**) 100 µm and (c–**f, h**) 20 µm.

In order to identify the brain regions affected by NiV and EBOV infection, ISH analyses were performed to assess viral infection and spread in different brain regions. NiV infection was observed in a scattered manner in various brain areas, including the cortex. Positive-stranded NiV RNA staining was predominantly found in the choroid plexus of the lateral ventricles and in meningeal cells of both BS models (Figs 4a–d, S5a–c). Occasionally, small dot-like staining was observed in neurons. Furthermore, via ISH, the distribution of EBOV infection was observed to be scattered in both BS models (Figs 4e–h, S5d–i), with fewer infected cells present in the affected regions compared to NiV at the evaluated time points. EBOV-NP staining was observed in neurons with inclusion bodies (Fig. 4e, f) and in choroid plexus cells (Fig. 4g, h). It is noteworthy that the choroid plexus appeared to be highly susceptible to both NiV (Fig. 4a–d) and EBOV (Fig. 4g, h) infection. This structure exhibited a relatively high level of infection compared to other brain regions. The data indicate that both EBOV and NiV are capable of infecting diverse cell types within the brain, irrespective of the immune status. This is evidenced by the fact that both IFNAR^−/−^-BS and C57BL/6J-BS were susceptible to infection.

### NiV can replicate in organotypic BS regardless of the immune status, whereas EBOV replication is limited

**Fig. 5.**
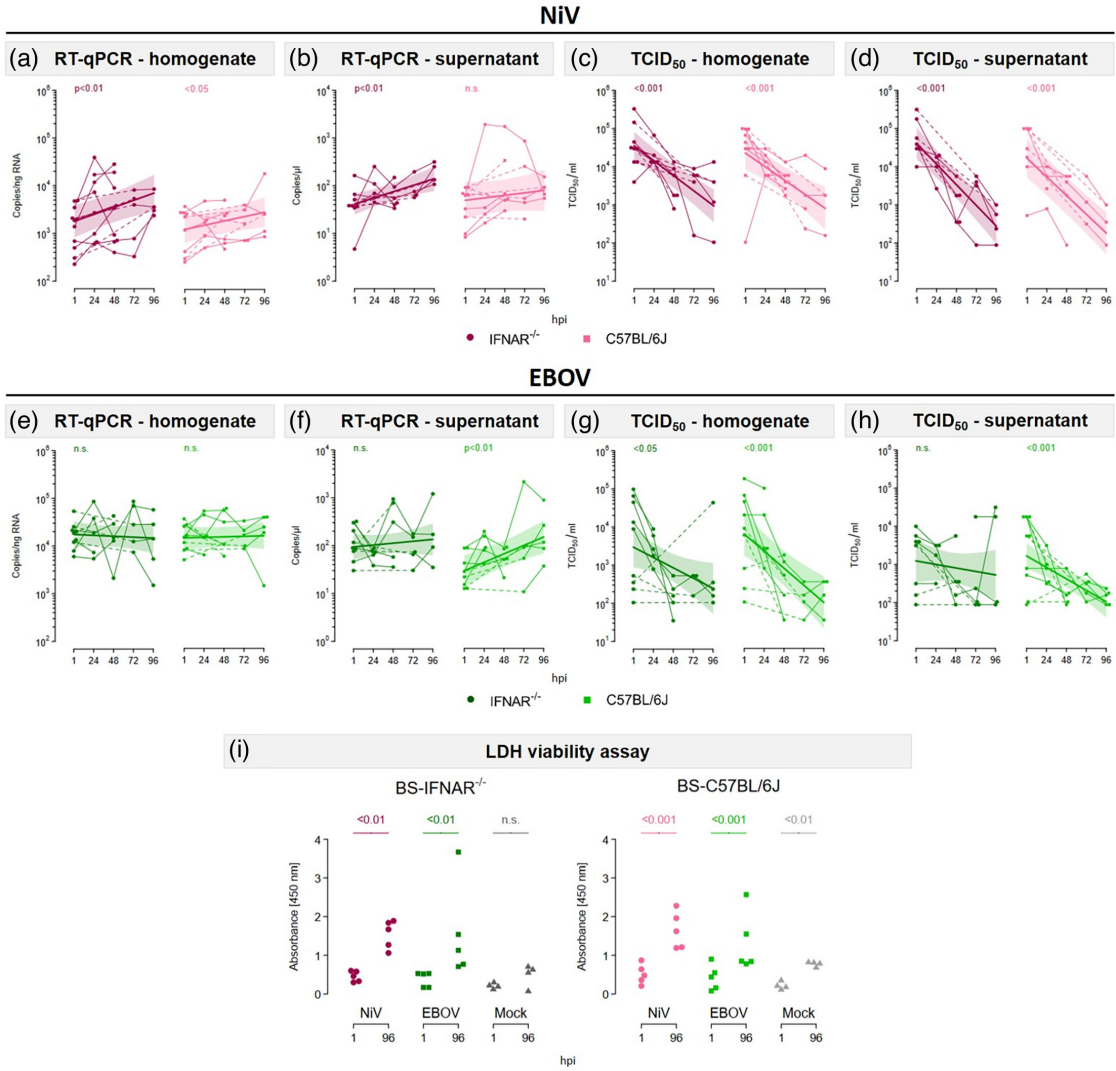
NiV and EBOV replication and tissue damage in organotypic BS. (a, b) Real-time qPCR to quantify (a) i.c. and (b) e.c. NiV RNA loads over time. (**c, d**) TCID_50_ titration of (b) homogenates and (c) supernatants at different time points. (**e, f**) Real-time qPCR to quantify (e) i.c. and (f) e.c. EBOV RNA load over time. (g, h) TCID_50_ titration of (e) homogenates and (f) supernatants. (**a–h**) Data are presented as individual values with connecting lines between related experiments. Dotted lines indicate time intervals that are discontinuous. Regression models were fitted to describe an exponential relationship between concentration and hpi, with log-normal errors. The curves depict the model predictions, with the coloured areas representing the approximate 95% confidence bands. The *P*-values are shown above the curves and refer to Wald t-tests of the growth rate coefficient (see the Methods section for details). Values below the LOD were defined as 0.5×LOD. (**i**) LDH viability assays to assess tissue damage after infection with NiV or EBOV. Individual values are shown. The *P*-values refer to Wald t-tests (see the Methods section for details). n.s., not significant.

Following an assessment of the susceptibility of the BS to EBOV or NiV infection, an additional analysis was conducted to determine the ability of the viruses to replicate efficiently in brain cells. To this end, real-time qPCR analysis and TCID_50_ assays were performed to evaluate the i.c. and e.c. viral RNA loads, as well as the release of infectious particles, respectively. Following NiV infection, an increase in i.c. viral RNA levels was observed in IFNAR^−/-^-BS (*P*<0.01) and in C57BL/6J-BS (*P*<0.05), indicating that NiV is capable of replicating in BS derived from both mouse strains (Fig. 5a). However, no clear difference in the growth rate of NiV was detected between the mouse strains. Furthermore, an increase in the e.c. viral RNA load was detected in BS-IFNAR^−/−^ (*P*<0.01), whereas no evident trend could be detected in C57BL/6J-BS (Fig. 5b). The number of infectious particles in the supernatant and homogenates exhibited a continuous decline over time in both, IFNAR^−/−^-BS and C57BL/6J-BS, commencing at 1 hpi (Fig. 5c, d), indicating that no release of infectious particles was detected over time. The continuous decrease in infectious particles in both supernatants and homogenates after NiV infection suggests that the input virus was slowly taken up or lost its infectivity. It is also plausible that infectious particles were not further released into the supernatant and that further virus propagation occurred by cell-to-cell spread. In contrast, following EBOV infection, no clear trend in viral RNA load over time was evident, except in the supernatants of IFNAR^−/−^-BS, where an increase in the e.c. viral RNA load was observed (*P*<0.01) (Fig. 5e, f). A reduction in infectious titres was observed over time via TCID_50_ assays, except for IFNAR^−/−^-BS supernatants, where no clear trend was identified (Fig. 5g, h). Nevertheless, the release of infectious particles was detected in individual experiments, indicating that EBOV infection can be productive in BS. Furthermore, LDH viability assays were conducted to assess tissue integrity following infection with either NiV or EBOV. A significant increase in LDH concentration in the supernatant was observed at 96 hpi compared to 1 hpi after either NiV or EBOV infection (Fig. 5i), indicating tissue damage. However, the available data did not allow for the detection of a clear difference in the growth rates of LDH release between mock and virus infection. These data suggest that while NiV can effectively replicate in BS, EBOV replication is limited. Moreover, no clear difference in the growth rate of these viruses was detected between genotypes.

### Adult organotypic BS release an array of cytokines and chemokines upon infection with NiV, EBOV and VSV

**Fig. 6.**
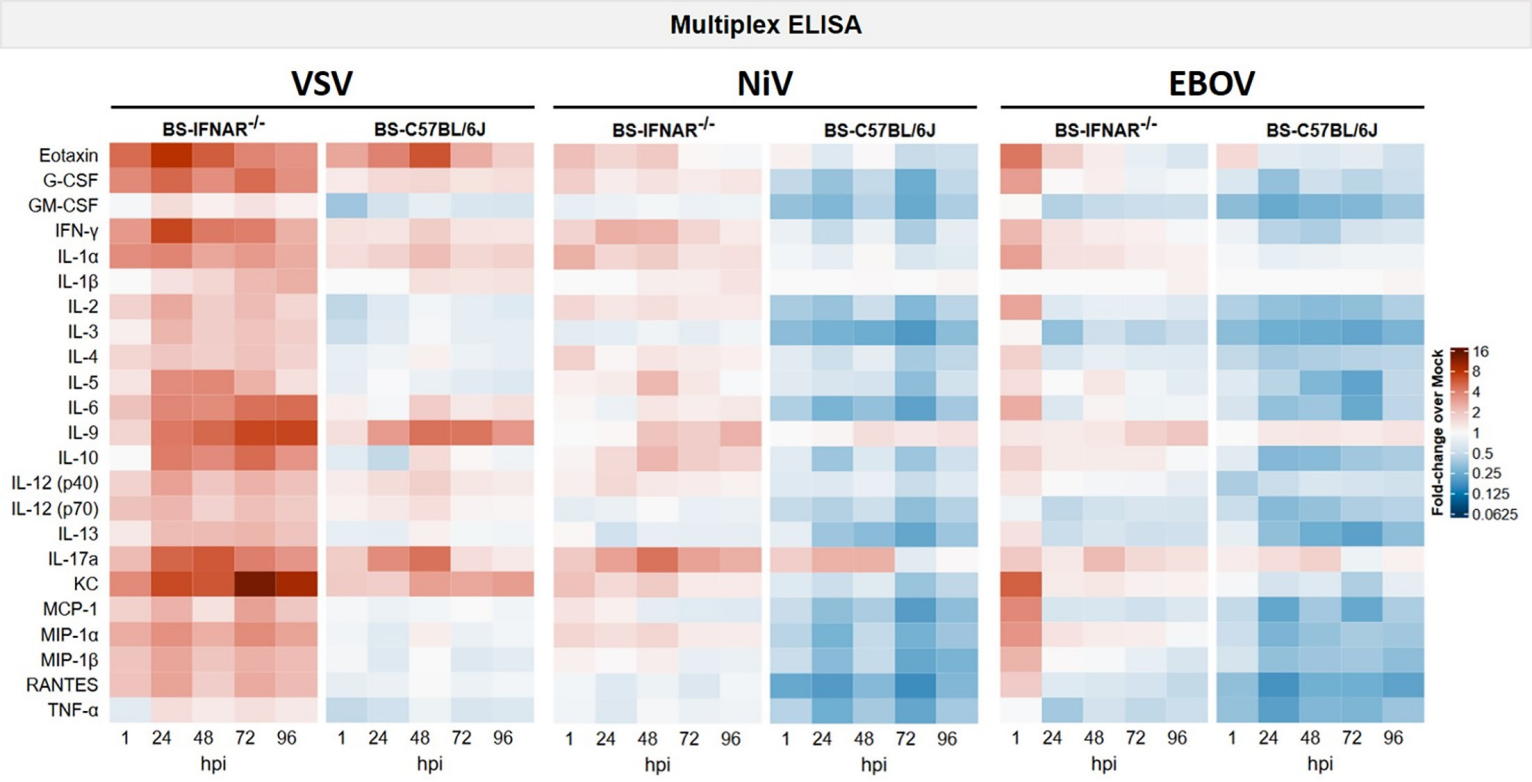
Brain-specific inflammatory responses to VSV infection, NiV and EBOV. Heatmaps show concentrations of cytokines and chemokines in the supernatant over time after infection with VSV, NiV or EBOV relative to mock-infected samples using multiplex ELISA based on the Luminex technology. The fold changes were obtained by dividing the mean value of each mediator at each time through the mean value of the respective mock sample. Averages were taken from three to five values.

To analyse the brain-specific inflammatory responses following infections with VSV, NiV or EBOV, the release of cytokines and chemokines was assessed via multiplex ELISA based on the Luminex technology. VSV infection was used as a proof-of-concept and positive control, given that VSV is a well-characterized neurotropic virus that exerts influence over the innate immune signalling. As mock-infected BS also released mediators, the release of mediators after infections is displayed as a fold change over mock (Fig. 6; for individual values, see Fig. S6–11). VSV infection resulted in the release of multiple cytokines and chemokines, many of which were found in significantly higher concentrations compared to mock infections (Table S1). These included the chemokines eotaxin, KC, MCP-1, MIP-1α, MIP-1β and RANTES; the pro-inflammatory cytokines IFN-γ, IL-1α, IL-6 and IL-17a and the anti-inflammatory cytokine IL-10 in IFNAR^−/−^-BS, among others. In contrast, in C57BL/6J-BS, only eotaxin, G-CSF, IFN-γ, IL-1α, IL-9, IL-12 (p40), IL-17a and KC were found to have significantly higher growth rates relative to mock. It is noteworthy that many mediators exhibited clear differences in the growth rates depending on the genotype of the BS (Table S2). Following NiV infection, multiple cytokines and chemokines were released, although to a lesser extent than in the case of VSV. This is in line with the overall lower viral replication for this virus. Furthermore, the differences between the mouse strains in relation to VSV were even more pronounced. The mediators that exhibited higher concentrations in IFNAR^−/−^-BS compared to mock were IL-17a, IFN-γ, IL-1α and IL-10. Conversely, mediators such as RANTES and MCP-1 were found in lower concentrations compared to mock. In the case of C57BL/6J-BS, most mediators were found in lower concentrations compared to mock. Following EBOV infection, an increase in the release of selected cytokines and chemokines, particularly in BS derived from IFNAR^−/−^ mice, was detected. However, many mediators were also found in lower concentrations. Interestingly, IL-9 and IL-17a were found in higher concentrations following infection with all three viruses, irrespective of the mouse strain.

## Discussion

Organotypic BS are typically isolated from embryonic or neonatal mice or rats and have been described to have better survival rates compared to adult slices. In this study, we successfully established an adult mouse organotypic BS culture for the purpose of studying the neurotropism of the highly pathogenic NiV and EBOV, as well as brain-specific inflammatory responses. The model is suitable for this purpose, as the presence of the major cell types of the brain was confirmed, which can act as potential target cells for either NiV or EBOV. Moreover, this study employed adult mice of varying ages. However, no conclusive evidence of age-related effects was found when examining the impact of age on the observed results (data not shown). Furthermore, the model’s suitability for neurotropic virus infection was confirmed, as evidenced by the efficient infection and replication of VSV in the slice model. The reduced replication in C57BL/6J-BS in comparison to that in IFNAR^−/−^-BS is consistent with the sensitivity of VSV to type I IFNs [50]. Previous *in vivo* findings have demonstrated that early and localized IFNAR triggering in the olfactory bulb of wt mice is a crucial factor for preventing viral spread over the entire CNS, thereby conferring survival benefits [51]. Moreover, the restricted release of infectious particles indicates that VSV mainly propagates via cell-to-cell spread in the BS, which has been demonstrated as a spreading strategy of VSV [52].

An inherent problem in the preparation process of the BS model is that axotomy induces neuronal death. The potential loss of specific vulnerable neuronal subsets indicates that these subsets cannot be evaluated as potential target cells. Moreover, given the lack of functionality in the vasculature, an analysis of haematogenous spread is not feasible. Nevertheless, evidence indicates that capillaries can survive in organotypic BS without any circulation. These capillaries are likely to express and secrete a cocktail of molecules that may influence other cells and nerve fibre innervation [53,54]. A further limitation of the model is the inability to analyse the role of infiltrating immune cells, although this, in turn, allows the study of brain-specific inflammatory responses.

The adult mouse organotypic BS model demonstrated susceptibility to NiV infection. However, in contrast to the inhibited replication following VSV infection of BS derived from wt mice, no eminent difference was observed between BS derived from both mouse strains following NiV infection. This suggests that type I IFN signalling may be a crucial factor in preventing VSV replication at the brain tissue level, but not for NiV. Moreover, the absence of infectious particle release, coupled with the extensive infection observed in selected regions, indicates that NiV propagation occurred via cell-to-cell spread. A significant number of animal viruses have evolved a multitude of mechanisms for viral spread. It is noteworthy that some of these mechanisms permit the direct transfer of viral particles or genomes from cell to cell, thereby circumventing the need for virus release [55]. Cell-to-cell spread has significant implications for pathogenesis, contributing to numerous pathogenic processes, including viral persistence and latency, therapy failure and resistance and immune evasion [55]. For example, NiV can propagate by cell-to-cell spread, whereby the membranes of adjacent cells fuse to form multinucleated cells [56]. This propagation strategy was also observed in this model. Nevertheless, it is conceivable that alternative strategies for cell-to-cell spread may have occurred because syncytial cell formation was limited. For instance, MV, which like NiV also belongs to the *Paramyxoviridae* family, has been observed to spread exclusively via the synapse in primary murine neurons. In contrast, the release of infectious particles has been shown to be a prerequisite for MV spread in primary murine fibroblasts. This underscores the impact of cell-type-specific differences on viral infection and pathogenesis [57]. Therefore, further research is required to gain a comprehensive understanding of the mechanisms employed by NiV.

The adult mouse BS model was also demonstrated to be susceptible to EBOV infection. Although EBOV replication was limited when whole slices were analysed via RT-qPCR, the presence of EBOV inclusions in neurons, as observed through IF and ISH, suggests that replication occurred in these cells. It is noteworthy that EBOV-NP staining was typically found in areas exhibiting astrogliosis, which is an increase in the number of astrocytes that occurs in response to pathology. This phenomenon gives rise to alterations in transcriptional regulation and in biochemical, morphological, metabolic and physiological remodelling, as well as the induction of immune responses. These changes are associated with beneficial functions but can also have adverse effects, leading to neurological sequelae such as encephalitis [58]. Furthermore, instances were observed where infectious particles were released, indicating that although viral replication was limited, infection could still be productive.

Infection with NiV and EBOV resulted in a decline in tissue integrity, as evidenced by an increase in LDH levels in the supernatants of both IFNAR^−/−^-BS and C57BL/6J-BS. However, the growth rate of LDH was not found to differ significantly from that of mock infections based on the available data.

Interestingly, an infection of the choroid plexus was frequently observed in this model following NiV and EBOV infection, where the infection was substantial. In addition, the ventricular system has been shown to be susceptible to infection in other animal models of NiV infection, including in ferrets [59] and in pigs [60]. Moreover, a recent study has demonstrated that EBOV can persist in the ventricular system of NHPs that have survived acute disease following treatment with monoclonal antibodies [61]. This persistence was associated with lethal disease outcomes and severe brain inflammation, highlighting a previously unidentified role for these cells in EBOV infection. Given that choroid plexus epithelial cells are tightly interconnected by tight junctions and represent the primary barrier components of the blood–CSF barrier, direct infection of these cells and subsequent cell death could result in a breach of the barrier function, thereby facilitating viral entry [62].

After analysing infection and replication of NiV and EBOV in BS, a further investigation was conducted into brain-specific inflammatory responses utilizing multiplex cytokine/chemokine ELISA with the Luminex technology. Mock-infected BS released cytokines and chemokines over time, which may represent basal levels. In a healthy brain, numerous mediators are released, including TNF-α, IFN-γ and IL-1β [63]. Basal IFN-γ is produced under homeostatic conditions in the meninges and performs specific functions, such as promoting neuronal survival and connectivity [64]. However, the release of certain mediators, exhibiting elevated concentrations such as IL-6, may also indicate an inflammatory response, potentially attributable to axotomy and cell death resulting from the cutting process, as well as during the culturing process. This may have contributed to the observed LDH release over time, including in mock infections. As a proof of concept, mediator release following VSV infection was evaluated. Many mediators were observed to be released at significantly higher levels in VSV-infected BS from both mouse strains compared to those in mock-infected BS. These included inflammatory mediators such as eotaxin, IFN-γ, IL-1α, IL-9, IL-12 (p40), IL-17a and KC. It is noteworthy that the release of mediators such as G-CSF, IL-13 and KC differed significantly between IFNAR^−/−^-BS and C57BL/6J-BS. These results indicate that the BS may respond to both injury and viral infections by releasing cytokines and chemokines, which could potentially lead to the recruitment of inflammatory cells and further inflammation *in vivo*.

After NiV infection of the BS, a number of cytokines and chemokines were released, albeit to a lesser extent than after VSV infection. Furthermore, pronounced differences were observed between BS derived from the two mouse strains. The concentrations of mediators including IL-17a, IFN-γ, IL-1α and IL-10 were significantly higher in infected IFNAR^−/−^-BS compared to mock infections. Conversely, the majority of mediators were released in diminished concentrations after infection of C57BL/6J-BS. These results demonstrate that NiV infection in IFNAR^−/−^-BS resulted in the release of numerous mediators, including pro-inflammatory cytokines and chemokines, as well as anti-inflammatory mediators with opposing functions, such as the induction of type 1, type 2 and type 3 immune responses. In humans infected with NiV, the release of inflammatory mediators including TNF-α, IFN-γ-induced protein 10 (IP-10) and IL-1β has been observed. These mediators have been shown to promote blood–brain barrier (BBB) disruption and the emergence of neurological symptoms observed in severe cases of NiV disease [65]. Moreover, it is frequently observed that inflammatory chemokines, such as IL-8 (mouse homologue KC), MIP-1α, MCP-1, RANTES and IP-10, are present in infectious CNS pathologies [66]. Pro-inflammatory cytokines are not only crucial mediators for viral clearance but also play a pivotal role in neuropathogenesis [67]. A study conducted on primary human brain endothelial cells revealed that infection with recombinant, wt NiV-Malaysia induces high levels of inflammatory cytokines such as IL-6, TNF-α, IL-8, eotaxin, MCP-1, MIP-1β and IP-10 [68]. These cytokines have the potential to increase BBB permeability and recruit immune cells *in vivo*. An increase in permeability may permit the infiltration of viruses, immune cells or immune cells carrying the virus into the CNS parenchyma. Neutrophils, macrophages and lymphocytes are among the inflammatory infiltrating immune cells found in human brain autopsies after NiV encephalitis [12].

EBOV infection of the BS resulted in an increase in specific mediators in comparison to mock infection. However, many other mediators were also found at reduced concentrations, including GM-CSF, IL-2, IL-3, IL-12 (p70), IL-13 and TNF-α. A recent study on immunocompetent mice infected with either wt-EBOV or mouse-adapted (ma)-EBOV revealed that both viruses disseminated and replicated widely in the animals, including in the brain. However, wt-EBOV induced viral tolerance associated with early type I IFN responses and reduced inflammation, in contrast to lethal ma-EBOV, which showed delayed type I IFN responses and elevated, unresolved inflammation. The pattern of induced mediators in the liver of mice infected with wt-EBOV was found to be similar to that released in C57BL/6J-BS after EBOV infection. Mediators such as TNF-α, IFN-γ, IL-6, IL-2, IL-4 and IL-1α were downregulated compared to mock. In contrast, the pattern observed following infection with ma-EBOV was similar to that observed in EBOV-infected IFNAR^−/−^-BS [69]. The results demonstrate that EBOV is capable of triggering the release of cytokines and chemokines in the BS models. This indicates that despite its limited replication, EBOV still was capable of inducing high levels of specific pro- and anti-inflammatory cytokines, suggesting that an infection in the brain parenchyma is sufficient to induce the release of these mediators. It is noteworthy that the release of many immune mediators was usually higher in IFNAR^−/−^-BS compared to that in C57BL/6J-BS following infections with VSV, NiV and EBOV. This could be in part due to higher viral replication in IFNAR^−/−^-BS compared to that in C57BL/6J-BS, but viral inhibition may also play an important role. For instance, VSV is known to suppress the IFN response mainly by inhibiting host cell transcription and translation. Additionally, the M protein has been demonstrated to target NFκB activation [70]. NiV and EBOV have been demonstrated to inhibit antiviral responses by multiple mechanisms, many of which target processes downstream of the IFNAR [71,72]. This may also contribute to the observed differences.

It is noteworthy that the concentrations of IL-9 and IL-17a increased in both IFNAR^−/−^-BS and C57BL/6J-BS following VSV, NiV and EBOV infections compared to those following mock infection. The IL-17a cytokine family has been proposed to play a crucial role in human inflammatory, autoimmune and neurodegenerative diseases, including Alzheimer’s disease, Parkinson’s disease and multiple sclerosis, likely by activating glial cells [73]. IL-17a has been demonstrated to impede the efficacy of T_H_1 (IFN-γ) responses, thereby facilitating pathogen persistence [74]. Furthermore, IL-17a has been linked to inducible nitric oxide synthase (iNOS)-mediated neuroinflammation, which has been shown to result in neuronal damage following Zika virus infection [75], as well as to BBB breakdown in multiple sclerosis [76]. IL-9 has been proposed as a key driver of immune responses in chronic inflammatory and autoimmune diseases at mucosal surfaces [77]. Furthermore, it has been shown to play a pathogenic role in an animal model of multiple sclerosis, namely experimental autoimmune encephalomyelitis, by augmenting T cell activation and differentiation and was significantly upregulated in the brain and spinal cord [78]. In light of the marked release of IL-17a and IL-9 in response to NiV and EBOV infections, it would be worthwhile to investigate their potential role in the neuropathology and persistence of these viruses.

Altogether, adult mouse organotypic BS can be used to study the neurotropism of highly pathogenic viruses such as NiV and EBOV as well as brain-specific inflammatory responses. This approach can contribute to the elucidation of the aetiology of viral pathology. Furthermore, it can be employed for preliminary assessment of prospective therapeutic modalities, thereby reducing the necessity for animal experimentation. Moreover, this approach is in accordance with the 3R principle that aims to reduce, refine and replace animal experiments. Our observations indicated that both EBOV and NiV were capable of infecting brain cells and inducing the release of multiple pro-inflammatory and anti-inflammatory cytokines, which might play a major role in CNS pathology. Lastly, the choroid plexus appears to be particularly susceptible to NiV and EBOV infection. This may contribute to the establishment of infection or facilitate persistence within the host.

## Supplementary material

10.1099/jgv.0.002038 Uncited Supplementary Material 1.

## Abbreviations

BBB: blood–brain barrier
BS: brain slices
BSL: biosafety level
CNS: central nervous system
CSF: cerebrospinal fluid
EBOV: Ebola virus
e.c.: extracellular
EVD: Ebola virus disease
GFAP: glial fibrillary acidic protein
GM-CSF: granulocytemacrophage colony-stimulating factor
HSV: herpes simplex virus
i.c.: intracellular
IFNAR-/-: type I IFN knockout
IP-10: IFN-γ-induced protein 10
ISH: *in-situ* hybridization
JEV: Japanese encephalitis virus
KC: keratinocyte-derived chemokine
LDH: lactate dehydrogenase
LOD: limit of detection
ma-EBOV: mouse-adapted EBOV
MBP: myelin basic protein
MCP-1: monocyte chemotactic protein-1
MIP: macrophage inflammatory protein
MTT: 3-(4,5-dimethylthiazol-2-yl)-2,5-diphenyltetrazolium bromide
MV: measles virus
NHP: non-human primate
NiV: nipah virus
RANTES: regulated upon activation normal T cell expressed and secreted
RT-qPCR: real-time quantitative PCR
TCID50: 50% tissue culture infectious dose
VSV: vesicular stomatitis virus
WNV: West Nile virus
