# Supplementary material for: Organotypic brain slices as a model to study the neurotropism of the highly pathogenic Nipah and Ebola viruses
Source: J Gen Virol. 2024 Oct 28;105(10):002038. doi: 10.1099/jgv.0.002038 (PMC12453409; doi:10.1099/jgv.0.002038)
Supplement: Uncited Supplementary Material 1. [file jgv-105-02038-s001.pdf]

1. Supplement

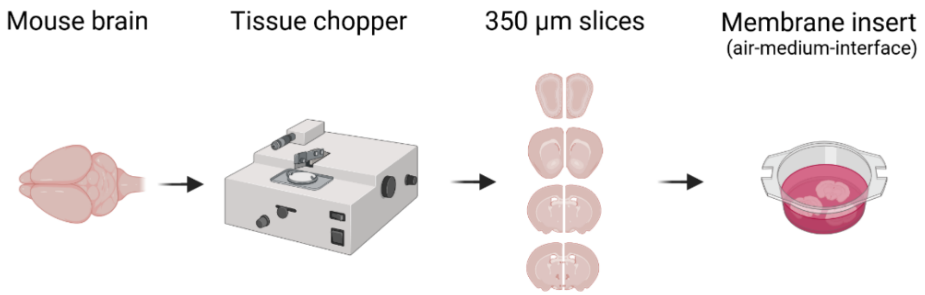

<u>*Suppl. Figure 1 Isolation and cultivation of organotypic murine brain slices*</u>*. To obtain organotypic brain slices (BS), the brain was isolated and bisected along the midline. The resulting hemibrains were sliced into 350 µm thick sections using a tissue chopper. After careful separation, four slices of different brain regions, namely olfactory bulb, frontal, middle, and caudal sections, were transferred onto a Millicell culture insert in a 6-well plate filled with 1 ml NG medium. Image was designed using Biorender.*

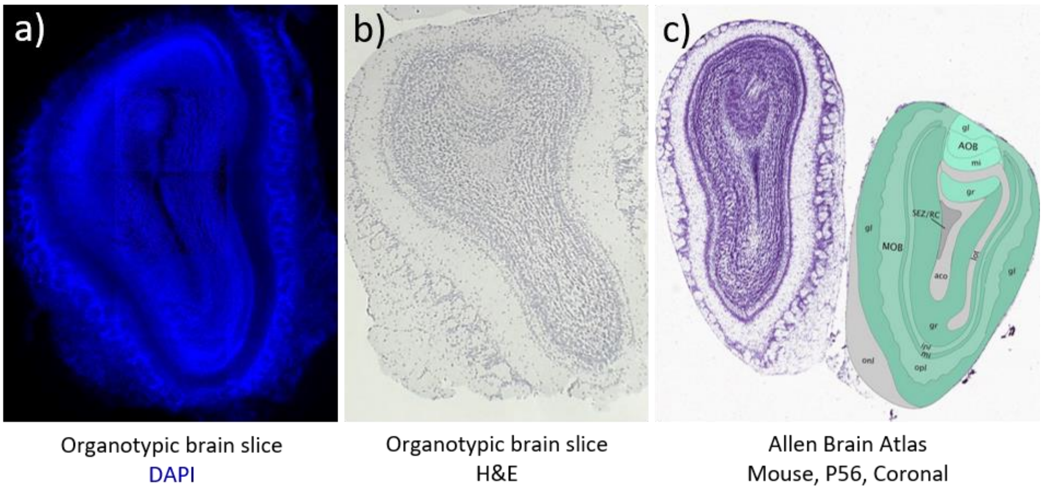

<u>*Suppl. Figure 2 Tissue architecture of the ex vivo organotypic brain slice culture model (BS).*</u> ***a)*** *Immunofluorescence image composed of several individual pictures taken with an Axiovert 200M at 100x magnification of a brain slice from the olfactory bulb stained with DAPI (blue) to visualize cell nuclei.* ***b)*** *Light microscopic image (Leica DM500) of the olfactory bulb stained with hematoxylin and eosin (H&E).* ***c)*** *Nissl staining (left) and anatomical annotations (right) of the murine olfactory bulb from the Allen Mouse Brain Atlas (mouse.brain-map.org).*

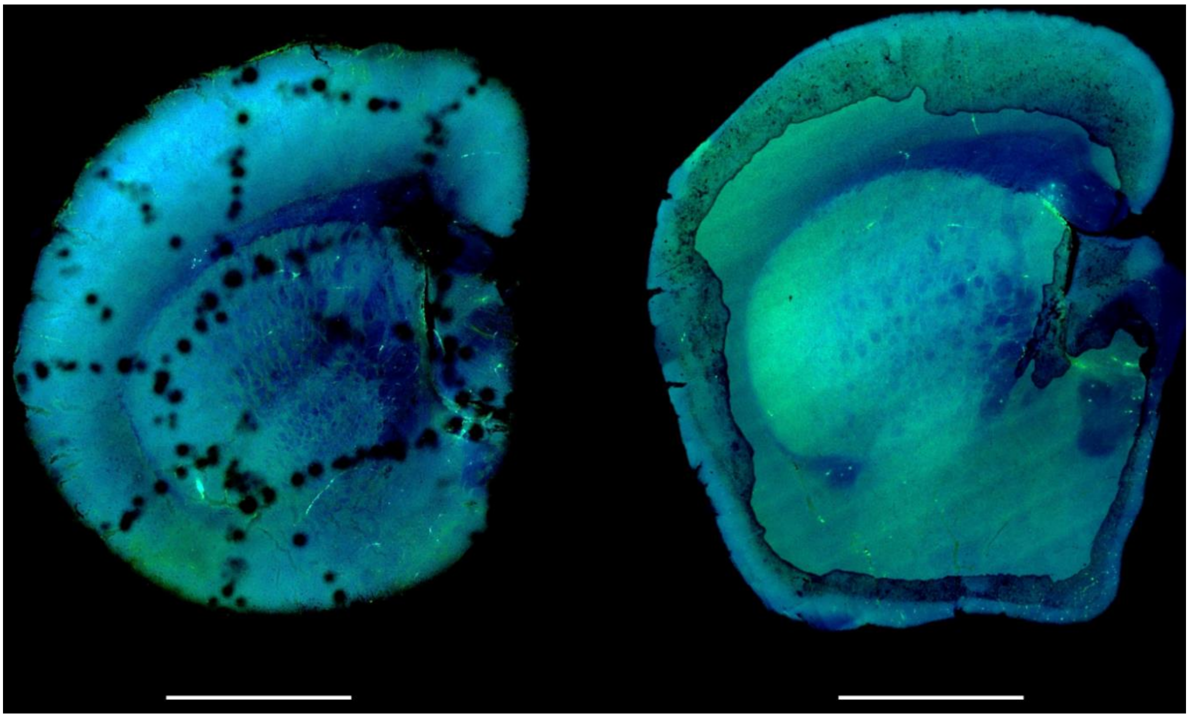

Suppl. Figure 3 Overview of vesicular stomatitis virus infection of murine adult organotypic brain slices
Immunofluorescence of two whole IFNAR$^{-/-}$-BS infected with VSV. Pictures were taken using the navigation function of a Leica THUNDER microscope. Black marks are mounting artifacts. Viral protein: VSV-N (green). Nuclear staining: DAPI (blue). Scale bars: 2 mm.

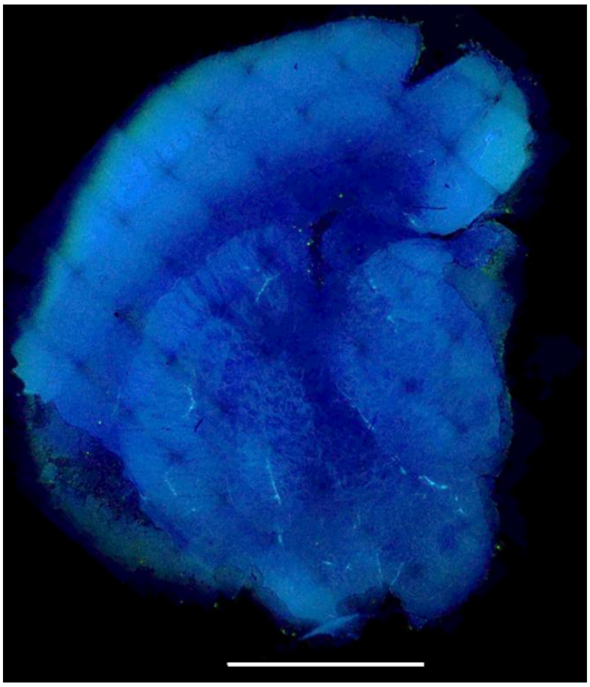

Suppl. Figure 4 Overview of C57BL/6J murine adult organotypic brain slices infected with vesicular stomatitis virus.
Immunofluorescence of a whole C57BL/6J-BS infected with VSV. Picture was taken using the navigation function of a Leica THUNDER microscope. Black marks are mounting artifacts. Viral protein: VSV-N (green). Nuclear staining: DAPI (blue). Scale bars: 2 mm.

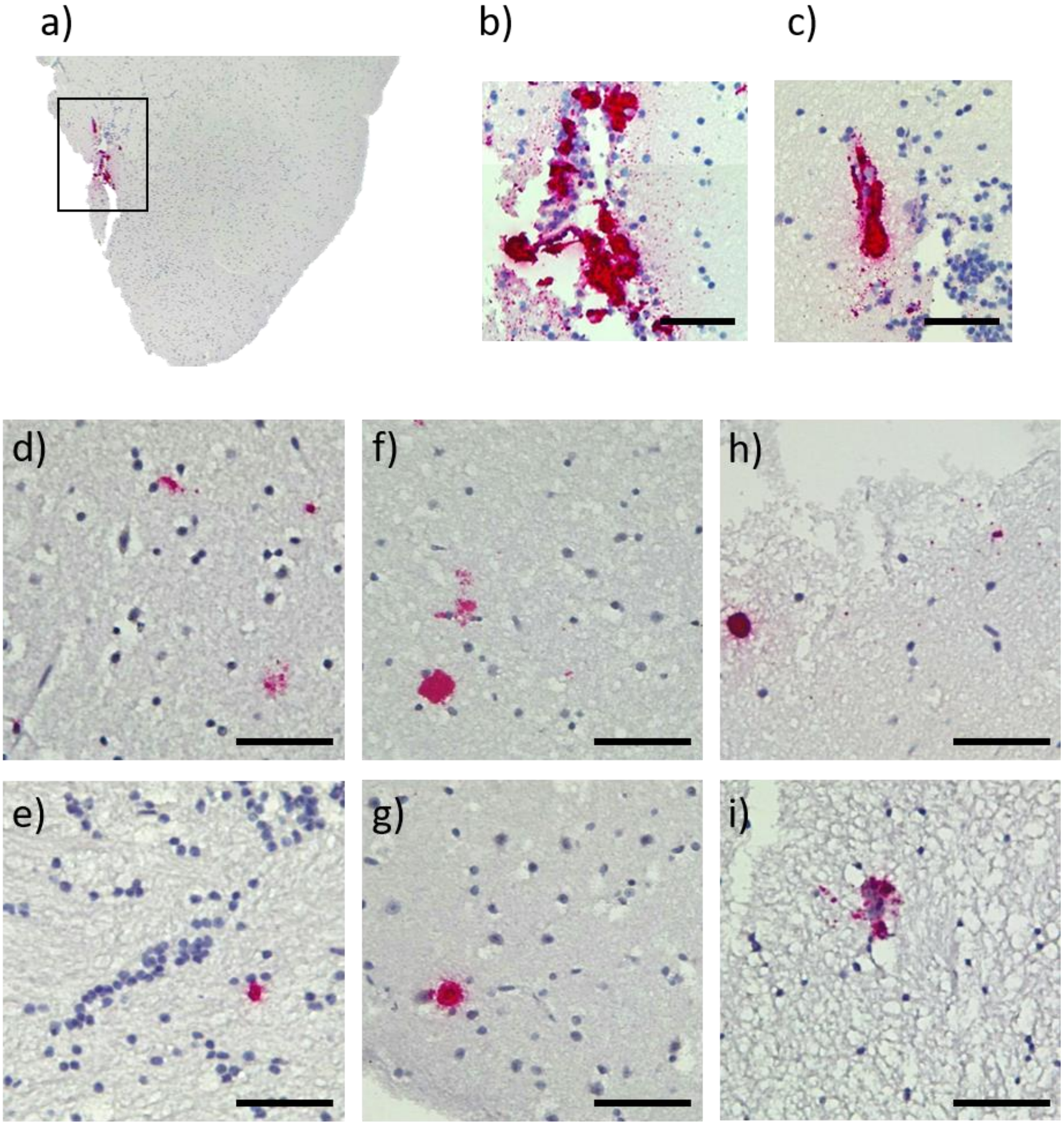

Suppl. Figure 5 Susceptibility of the C57BL/6J organotypic brain slice culture (BS) to NiV and EBOV infection. ***a-c)*** *In situ hybridization of BS-C57BL/6J at 96 hpi displaying staining for viral mRNA (V-Nipah-StrainM/B.-N-Probe). a) Low magnification picture displaying staining in the lateral ventricle. b-c) higher magnification of a).* ***d-i)*** *In situ hybridization of EBOV-infected BS-C57BL/6J showing positive staining for viral RNA (V-EBOV-VP35-GP-probe) at d-e) 24 hpi, f-g) 48 hpi, and h-i) 96 hpi. Scale bars: 20 µm. M/B: Malaysia/Bangladesh. Hpi: hours post infection.*

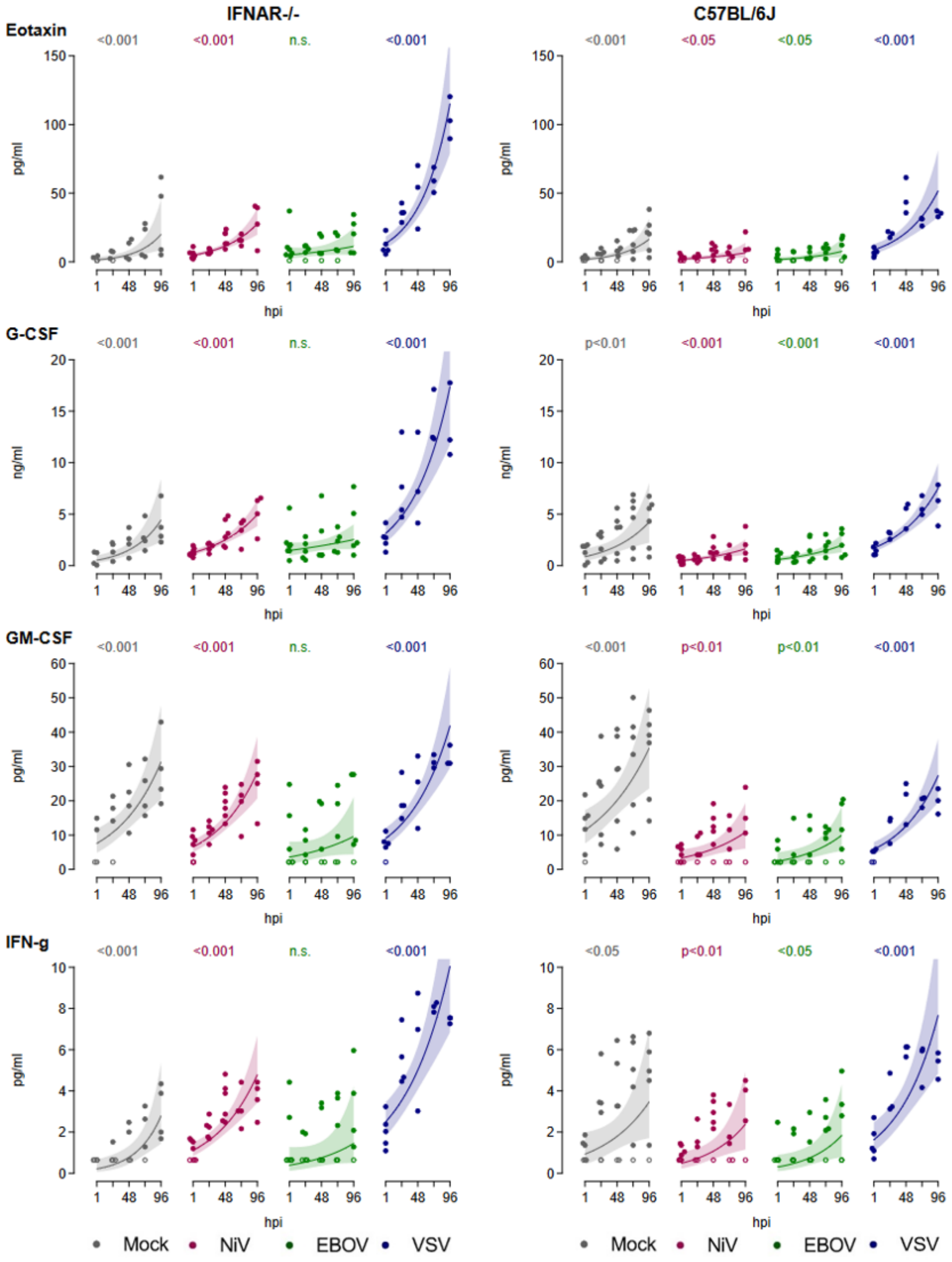

Suppl. Figure 6 Release of Eotaxin, G-CSF, GM-CSF, and IFN-γ after mock, NiV, EBOV, and VSV infection. The release was analyzed using multiplex ELISA based on the Luminex technology. The data are presented as individual values. A regression model was fitted to describe an exponential relationship between concentration and hpi, with log-normal errors. The curves depict the model predictions, with the colored areas representing the approximate 95% confidence bands. The p-values are shown above the curves and refer to Wald t-tests of the growth rate coefficient (see methods section for details). Values at the lower limit of quantification are shown as open circles. The values were taken as left-censored in the regression model. n.s.: not significant.

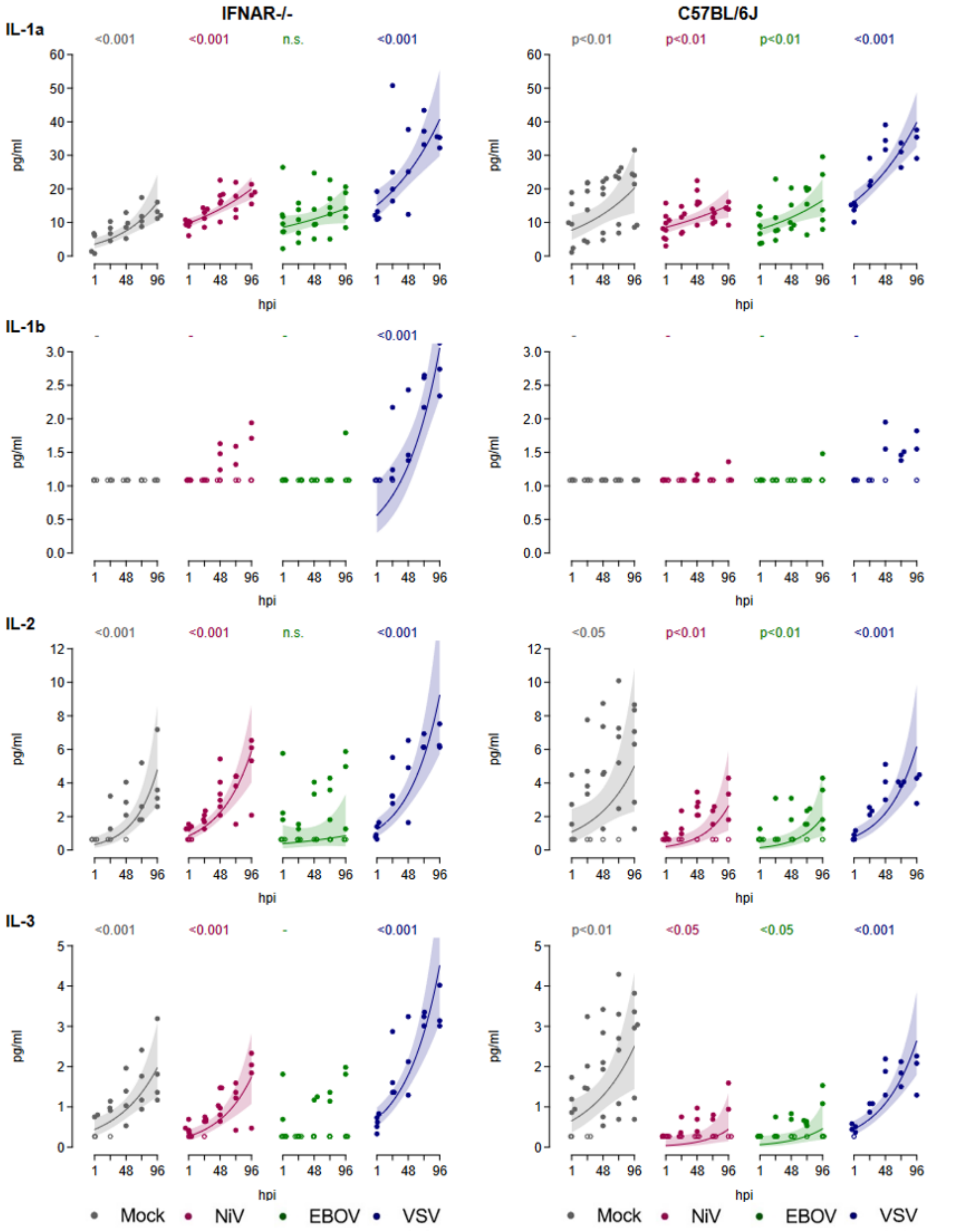

Suppl. Figure 7 Release of IL-1α, IL-1β, IL-2, and IL-3 after mock, NiV, EBOV, and VSV infection. The release was analyzed using multiplex ELISA based on the Luminex technology. The data are presented as individual values. A regression model was fitted to describe an exponential relationship between concentration and hpi, with log-normal errors. The curves depict the model predictions, with the colored areas representing the approximate 95% confidence bands. The p-values are shown above the curves and refer to Wald t-tests of the growth rate coefficient (see methods section for details). Values at the lower limit of quantification (LLOQ) are shown as open circles. These values were taken as left-censored in the regression model. In instances with >70% censored data, it was not possible to obtain a meaningful estimate of the trend. Consequently, the p-value was not provided, and no model curve is shown. n.s.: not significant.

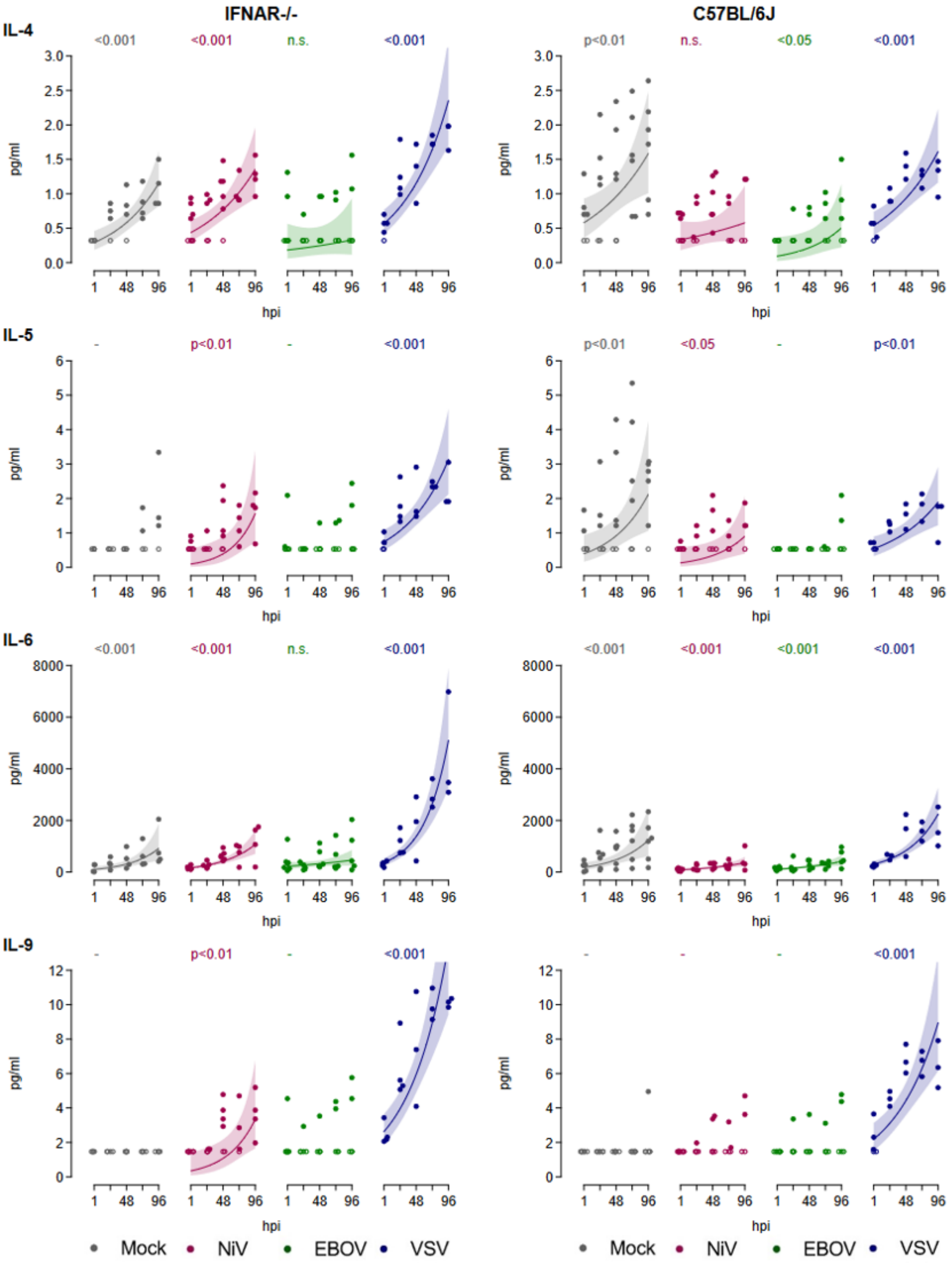

<u>Suppl. Figure 8 Release of IL-4, IL-5, IL-6, and IL-9 after mock, NiV, EBOV, and VSV infection</u>. The release was analyzed using multiplex ELISA based on the Luminex technology. The data are presented as individual values. A regression model was fitted to describe an exponential relationship between concentration and hpi, with log-normal errors. The curves depict the model predictions, with the colored areas representing the approximate 95% confidence bands. The p-values are shown above the curves and refer to Wald t-tests of the growth rate coefficient (see methods section for details). Values at the lower limit of quantification (LLOQ) are shown as open circles. These values were taken as left-censored in the regression model. In instances with >70% censored data, it was not possible to obtain a meaningful estimate of the trend. Consequently, the p-value was not provided, and no model curve is shown. n.s.: not significant.

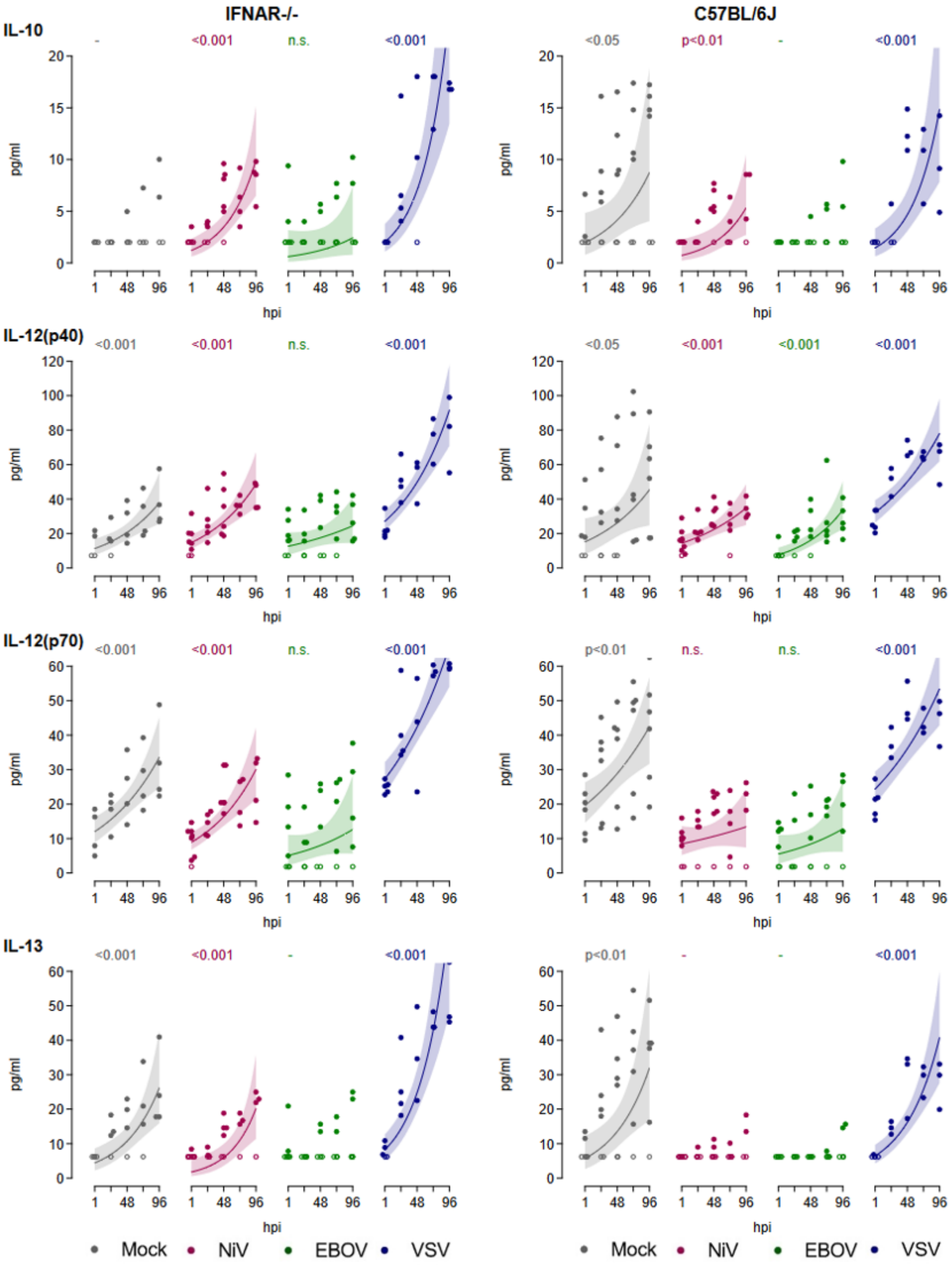

Suppl. Figure 9 Release of IL-10, IL-12 (p40), IL-12 (p70), and IL-13 after mock, NiV, EBOV, and VSV infection. *The release was analyzed using multiplex ELISA based on the Luminex technology. The data are presented as individual values. A regression model was fitted to describe an exponential relationship between concentration and hpi, with log-normal errors. The curves depict the model predictions, with the colored areas representing the approximate 95% confidence bands. The p-values are shown above the curves and refer to Wald t-tests of the growth rate coefficient (see methods section for details). Values at the lower limit of quantification (LLOQ) are shown as open circles. These values were taken as left-censored in the regression model. In instances with >70% censored data, it was not possible to obtain a meaningful estimate of the trend. Consequently, the p-value was not provided, and no model curve is shown. n.s.: not significant.*

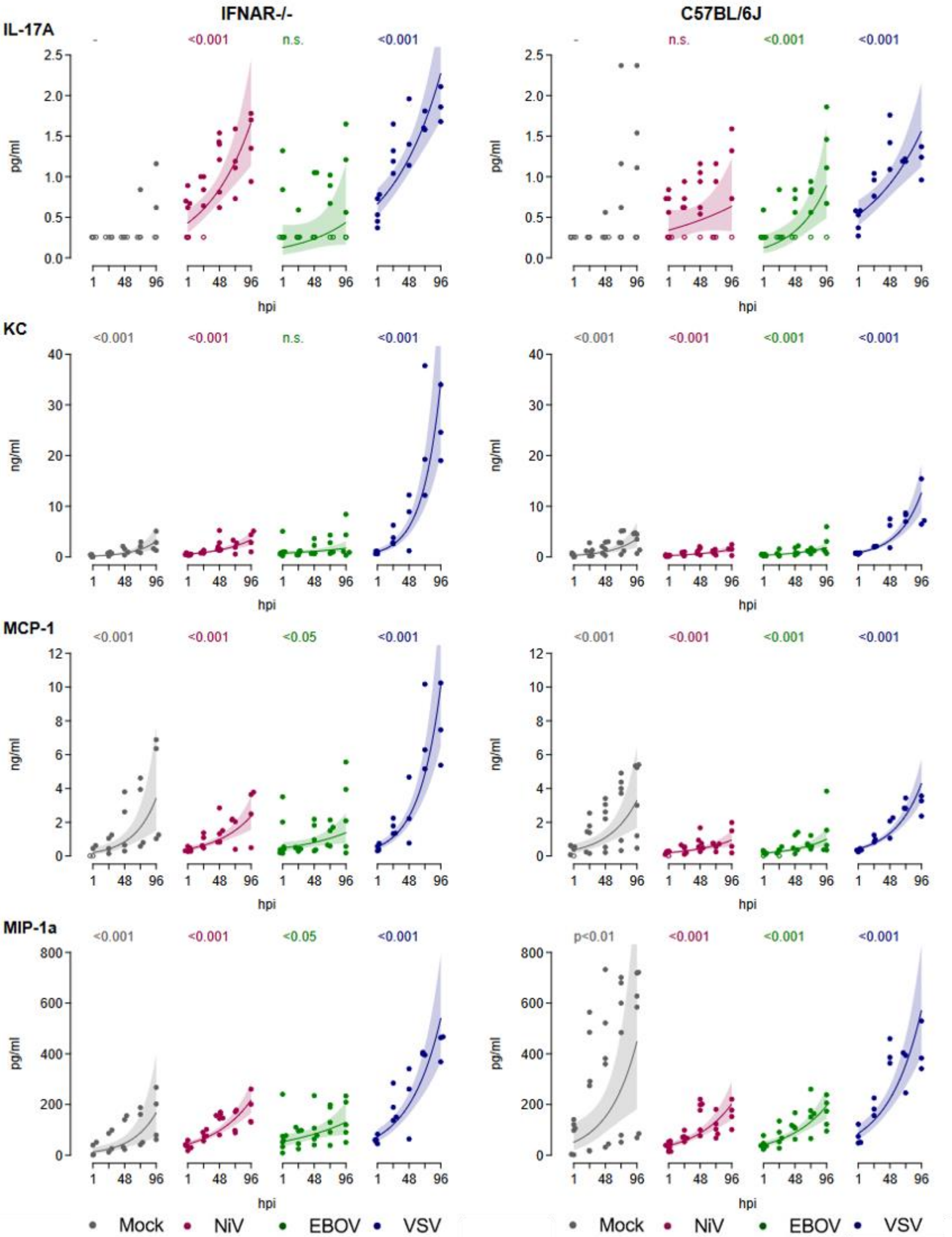

Suppl. Figure 10 Release of IL-17a, KC, MCP1, and MIP-1α after mock, NiV, EBOV, and VSV infection. The release was analyzed using multiplex ELISA based on the Luminex technology. The data are presented as individual values. A regression model was fitted to describe an exponential relationship between concentration and hpi, with log-normal errors. The curves depict the model predictions, with the colored areas representing the approximate 95% confidence bands. The p-values are shown above the curves and refer to Wald t-tests of the growth rate coefficient (see methods section for details). Values at the lower limit of quantification (LLOQ) are shown as open circles. These values were taken as left-censored in the regression model. In instances with >70% censored data, it was not possible to obtain a meaningful estimate of the trend. Consequently, the p-value was not provided, and no model curve is shown. n.s.: not significant.

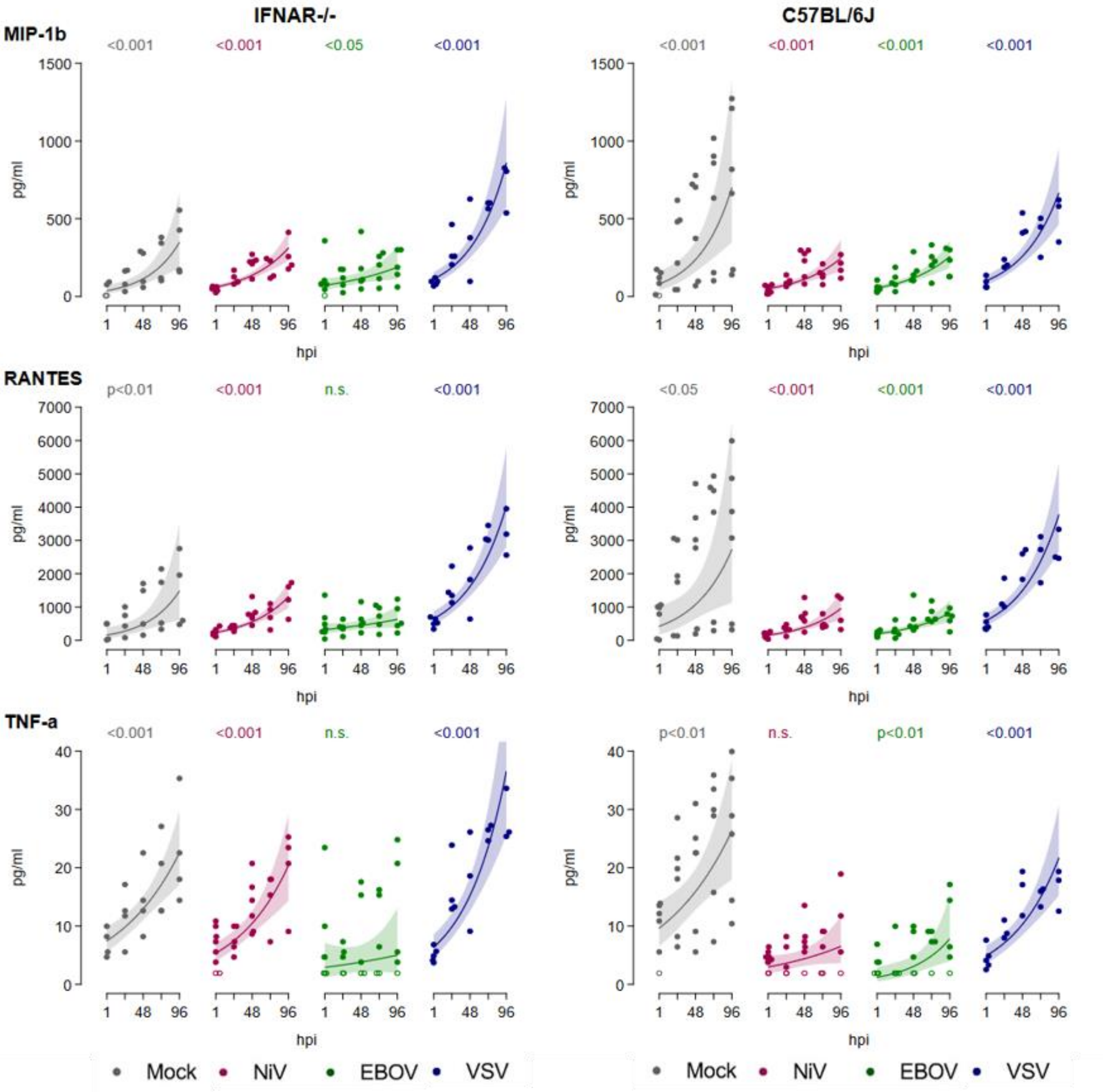

Suppl. Figure 11 Release of MIP-1β, RANTES, and TNF-α after mock, NiV, EBOV, and VSV infection. The release was analyzed using multiplex ELISA based on the Luminex technology. The data are presented as individual values. A regression model was fitted to describe an exponential relationship between concentration and hpi, with log-normal errors. The curves depict the model predictions, with the colored areas representing the approximate 95% confidence bands. The p-values are shown above the curves and refer to Wald t-tests of the growth rate coefficient (see methods section for details). Values at the lower limit of quantification (LLOQ) are shown as open circles. These values were taken as left-censored in the regression model. n.s.: not significant.

Mediator	NiV vs Mock		EBOV vs Mock		VSV vs Mock	
	IFNAR$^{-/-}$	C57BL/6J	IFNAR$^{-/-}$	C57BL/6J	IFNAR$^{-/-}$	C57BL/6J
**Eotaxin**	n.s	<0.05	n.s	n.s	<0.001	<0.001
**G-CSF**	n.s	<0.001	n.s	<0.01	<0.001	<0.05
**GM-CSF**	n.s	<0.001	<0.001	<0.001	n.s	n.s
**IFN-g**	<0.05	n.s	n.s	<0.05	<0.001	<0.05
**IL-1a**	<0.05	n.s	n.s	n.s	<0.001	<0.001
**IL-1b**	n.s	n.s	NA	NA	n.s	NA
**IL-2**	n.s	<0.01	<0.05	<0.001	<0.05	n.s
**IL-3**	n.s	<0.001	<0.001	<0.001	<0.01	n.s
**IL-4**	n.s	<0.001	n.s	<0.001	<0.001	n.s
**IL-5**	n.s	<0.01	n.s	<0.001	<0.001	n.s
**IL-6**	n.s	<0.001	n.s	<0.001	<0.001	n.s
**IL-9**	n.s	n.s	n.s	n.s	n.s	<0.001
**IL-10**	<0.05	<0.01	n.s	<0.001	<0.001	n.s
**IL-12(p40)**	n.s	n.s	n.s	<0.01	<0.001	<0.01
**IL-12(p70)**	n.s	<0.001	<0.01	<0.001	<0.05	n.s
**IL-13**	n.s	<0.001	<0.01	<0.001	<0.001	n.s
**IL-17A**	<0.001	n.s	n.s	n.s	<0.001	<0.001
**KC**	n.s	<0.05	n.s	n.s	<0.001	<0.001
**MCP-1**	n.s	<0.001	n.s	<0.001	<0.01	n.s
**MIP-1a**	n.s	<0.05	n.s	<0.05	<0.001	n.s
**MIP-1b**	n.s	<0.001	n.s	<0.001	<0.01	n.s
**RANTES**	n.s	<0.001	n.s	<0.001	<0.01	n.s
**TNF-a**	n.s	<0.001	<0.001	<0.001	n.s	n.s

Suppl. Table 1 Comparison of the growth rates of released mediators between mock and virus infections. A comparative analysis was conducted between a virus and a mock infection (NiV, EBOV, VSV) within the context of a specific genotypic classification: IFNAR$^{-/-}$-BS or C57BL/6J-BS.The p-values refer to Wald t-tests of the growth rate coefficient (see Methods section for details). n.s.: not significant.

Mediator	IFNAR$^{-/-}$ vs C57BL/6J			
	Mock	NiV	EBOV	VSV
**Eotaxin**	n.s	<0.001	n.s	n.s
**G-CSF**	n.s	<0.001	n.s	<0.05
**GM-CSF**	n.s	<0.001	n.s	n.s
**IFN-g**	<0.05	<0.05	n.s	n.s
**IL-1a**	<0.05	n.s	n.s	n.s
**IL-1b**	NA	n.s	NA	NA
**IL-2**	n.s	<0.01	n.s	n.s
**IL-3**	n.s	<0.01	n.s	n.s
**IL-4**	<0.01	<0.05	n.s	n.s
**IL-5**	<0.01	n.s	n.s	n.s
**IL-6**	n.s	<0.001	n.s	n.s
**IL-9**	n.s	n.s	n.s	n.s
**IL-10**	<0.001	n.s	n.s	n.s
**IL-12(p40)**	n.s	n.s	n.s	n.s
**IL-12(p70)**	n.s	<0.05	n.s	n.s
**IL-13**	n.s	<0.01	n.s	<0.05
**IL-17A**	n.s	<0.01	n.s	n.s
**KC**	n.s	<0.01	n.s	<0.05
**MCP-1**	n.s	<0.01	n.s	n.s
**MIP-1a**	<0.001	n.s	n.s	n.s
**MIP-1b**	<0.01	n.s	n.s	n.s
**RANTES**	<0.05	n.s	n.s	n.s
**TNF-a**	n.s	<0.001	n.s	n.s

Suppl. Table 2 Comparison of growth rates of released mediators between genotypes. A comparative analysis was conducted between the genotypes (IFNAR$^{-/-}$-BS, C57BL/6J-BS) within the context of a specific infection (mock, NiV, EBOV, VSV). The p-values refer to Wald t-tests of the growth rate coefficient (see Methods section for details). n.s.: not significant.
